# Comparing the Role of ROS and RNS in the Thermal Stress Response of Two Cnidarian Models, *Exaiptasia diaphana* and *Galaxea fascicularis*

**DOI:** 10.3390/antiox12051057

**Published:** 2023-05-06

**Authors:** Talisa Doering, Justin Maire, Wing Yan Chan, Alexis Perez-Gonzalez, Luka Meyers, Rumi Sakamoto, Isini Buthgamuwa, Linda L. Blackall, Madeleine J. H. van Oppen

**Affiliations:** 1 School of Biosciences, The University of Melbourne, Parkville, VIC 3010, Australia; 2 Department of Microbiology and Immunology, The University of Melbourne at the Peter Doherty Institute of Infection and Immunity, Parkville, VIC 3010, Australia; 3 Melbourne Cytometry Platform, The University of Melbourne, Parkville, VIC 3010, Australia; 4 Australian Institute of Marine Science, Townsville, QLD 4810, Australia

**Keywords:** *Galaxea fascicularis*, *Exaiptasia diaphana*, reactive oxygen species, nitric oxide, coral bleaching, superoxide dismutase, catalase, nitric oxide synthase

## Abstract

Coral reefs are threatened by climate change, because it causes increasingly frequent and severe summer heatwaves, resulting in mass coral bleaching and mortality. Coral bleaching is believed to be driven by an excess production of reactive oxygen (ROS) and nitrogen species (RNS), yet their relative roles during thermal stress remain understudied. Here, we measured ROS and RNS net production, as well as activities of key enzymes involved in ROS scavenging (superoxide dismutase and catalase) and RNS synthesis (nitric oxide synthase) and linked these metrics to physiological measurements of cnidarian holobiont health during thermal stress. We did this for both an established cnidarian model, the sea anemone *Exaiptasia diaphana*, and an emerging scleractinian model, the coral *Galaxea fascicularis*, both from the Great Barrier Reef (GBR). Increased ROS production was observed during thermal stress in both species, but it was more apparent in *G. fascicularis*, which also showed higher levels of physiological stress. RNS did not change in thermally stressed *G. fascicularis* and decreased in *E. diaphana*. Our findings in combination with variable ROS levels in previous studies on GBR-sourced *E. diaphana* suggest *G. fascicularis* is a more suitable model to study the cellular mechanisms of coral bleaching.

## 1. Introduction

Climate change is threatening the persistence of reef-building corals and coral reefs, because it causes both a gradual increase in sea surface temperatures (SSTs) as well as an increase in the frequency, intensity, and duration of summer heatwaves. A combination of above-normal SSTs and irradiance during heatwaves is the primary cause of mass coral bleaching [1], the loss of the algal photosymbionts (family Symbiodiniaceae) from the coral host tissues. Symbiodiniaceans provide a significant amount of photosynthetically fixed carbon to their coral host, which can account for up to 90% of the host’s carbon consumption and up to 150% of its daily respiratory requirements [2,3]. As a consequence, bleaching often leads to coral starvation and death, as well as to the degradation of coral reefs [4].

**Figure 1:**
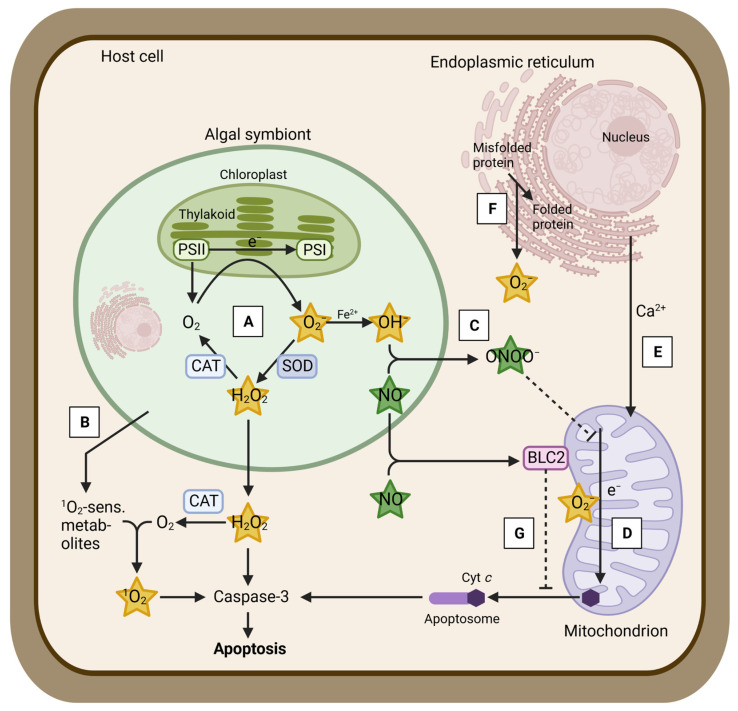
Main mechanisms hypothesized to be involved in the coral bleaching cascade. Step A: Generation of various reactive oxygen species (ROS) in response to damage to the symbiodiniacean photosynthetic machinery due to thermal and light stress. Formation of superoxide (O_2_^−^) from photosynthetic O_2_ due to the inhibition of the electron transfer from PSII to PSI (Mehler reaction). Transformation of O_2_^−^ to hydrogen peroxide (H_2_O_2_) by superoxide dismutase (SOD) and further to O_2_ by catalase (CAT). H_2_O_2_ may be released to the host cells via aquaporins. Formation of hydroxyl radicals (OH^−^) from O_2_^−^ if transition metal ions (Fe^2+^) are present (Haber–Weiss reaction). Step B: Generation of singlet oxygen (^1^O_2_) from photosynthetic O_2_ and ^1^O_2_-sensitizing metabolites, previously released by the algal symbiont. Step C: Formation of peroxynitrite (ONOO^−^) from nitric oxide (NO) and OH^−^ disrupts electron transport within mitochondria. Step D: Acceleration of ROS production within mitochondria due to increased respiration and inhibition of the electron transport. Step E: Calcium (Ca^2+^) release from the endoplasmic reticulum to the mitochondria and subsequent release of cytochrome c (Cyt c) from mitochondria. Step F: Further ROS production in the endoplasmic reticulum as a consequence of enhanced protein repair and re-folding. Step G: Increased ROS, NO, or mitochondrial uptake of Ca^2+^ impedes the mitochondrial B-cell lymphoma 2 protein (BCL2) to prevent the release of Cyt c. Host cell apoptosis in response to Cyt c binding to the apoptosome and inducing caspase activity. Figure adapted with permission from Oakley and Davy [5]; published by Springer Nature, 2018. Created with BioRender.com (accessed on 23 April 2023).

There are several hypotheses describing the cellular mechanisms underpinning coral bleaching. The oxidative stress hypothesis states that elevated irradiance and temperature cause an overproduction of toxic reactive oxygen species (ROS, Figure 1) [5,6,7]. Even under non-stress conditions, various types of ROS are continuously produced during photosynthesis in the algal symbionts, i.e., by electron transfer from photosystem II to photosystem I or by reducing state triplet oxygen [8]. Singlet oxygen (^1^O_2_) is generated in the reaction system of photosystem II [9], and superoxide (O_2_^−^) is formed at the electron acceptor site of the photosystem I via the direct reduction of oxygen [10,11] (Figure 1, step A). O_2_^−^ is reduced to H_2_O_2_ by superoxide dismutase (SOD) [12,13]. Hydroxyl radicals (OH^−^) are formed via the Haber–Weiss reaction, which reduces H_2_O_2_ by O_2_^−^ if transition metal ions are present [14]. All types of ROS produced under regular physiological conditions are promptly scavenged by the antioxidant defense system, which consists of various enzymatic and non-enzymatic mechanisms within the holobiont. Scavenging enzymes include SOD and catalase (CAT) [15,16,17,18] (Figure 1, steps A,B). For instance, SOD is abundant in corals and is expressed in both host [19,20,21,22] and symbiodiniacean [23,24] compartments. During elevated irradiance and light levels, ROS increase in the coral, mainly due to the disruption of the photosymbionts’ photosystem II and its associated D1 protein, Calvin–Benson–Bassham cycle reactions (reductive pentose phosphate cycle), and thylakoid membranes [6,25,26] (Figure 1, step A). Excess ROS may overwhelm symbiodiniacean antioxidant defense mechanisms, diffuse into the host cells, where they cause damage to macromolecules (e.g., DNA), and trigger a cellular cascade that leads to bleaching [6,7] (Figure 1). One of the most relevant ROS during bleaching is hydrogen peroxide (H_2_O_2_), as it may diffuse to host cells via aquaporins [27], may be temporally more stable than other ROS (1–3 min in mammalian cells and 10 s in *Arabidopsis* guard cells [28]), and might act as a signaling molecule between the algal symbionts and the coral host [11,29]. 

Other studies have posed that bleaching is initiated when the coral host cannot meet the CO_2_ demands of symbiodiniaceans, which may grow faster under elevated temperatures [30,31]. This is believed to damage Calvin–Benson cycle reactions, causing an accelerated production of ROS that may diffuse into the host cells and trigger bleaching. Another hypothesis postulates that thermal stress alters nutrient cycling between the algal symbiont and its coral host [32]. As elevated temperatures enhance host respiration and catabolism, more ammonium becomes available for symbiodiniaceans, releasing them from their nitrogen-limited state and boosting their growth. Consequently, the algal symbionts use most photosynthate for their own proliferation instead of translocating it to their coral host [33]. Higher symbiodiniacean growth triggers an imbalance in the N:P ratio, altering the composition of the thylakoid membranes [34] and resulting in an overproduction of ROS that causes bleaching. Given that all of the main bleaching hypotheses describe the overproduction of ROS, it can be assumed that ROS are major drivers of the bleaching cascade.

In addition to ROS, reactive nitrogen species (RNS), such as nitric oxide, have been proposed to play a role in coral bleaching [6,7] (Figure 1, step C). Overall, nitric oxide is known to be an essential cellular signaling molecule for microbes within the coral holobiont involved in apoptosis and immunity [5,6,35]. In contrast to most ROS, nitric oxide is able to pass membranes and to enter coral host cells due to its lipophilic nature [5]. Under elevated temperatures, increased levels of nitric oxide and/or increased activity of nitric oxide-producing enzymes (i.e., nitric oxide synthase (NOS)) have been measured in symbiodiniacean cultures [36] and in the cnidarian model, the sea anemone *Exaiptasia diaphana* (in both the host and symbiodiniacean compartments), and these have been correlated with bleaching, symbiodiniacean photoinhibition, and death [37,38,39]. Hawkins et al. [35,36,40,41,42] suggested that nitric oxide plays a role in inducing host apoptotic pathways in response to symbiont dysfunction during bleaching. Signaling pathways of both ROS and RNS may interact [7,37,38], but their relative contributions during bleaching remain largely unknown. One suggested interaction of both pathways is the reaction of nitric oxide with O_2_^−^ that generates peroxynitrite (ONOO^−^), which disrupts electron transport within mitochondria [5] (Figure 1 step C). Further, nitric oxide production, measured by NOS activity, played a more prominent role under acute thermal stress in freshly isolated symbiodiniaceans from the coral *Pocillopora acuta* than oxidative stress based on the presence of H_2_O_2_ and OH^−^ [43]. Further studies are required to disentangle the relative roles of ROS and RNS during coral bleaching.

To understand the contribution of either ROS or RNS during thermal coral bleaching, previous studies have made use of cnidarian models to measure some of these reactive species during thermal stress, such as in the sea anemone *Exaiptasia diaphana* (Actiniaria order, Aiptasiidae family; Rapp 1829) [37,44,45]. For *E. diaphana* from the Great Barrier Reef (GBR), recent studies demonstrated that bleaching was not accompanied by elevated levels of ROS [45,46], which did not support the oxidative stress theory of coral bleaching. Since *E. diaphana* has been a widely used model worldwide, we wanted to confirm these findings in GBR-sourced *E. diaphana* and expand upon them by studying the contribution of RNS during thermal stress. We also included an emerging scleractinian model, the coral *Galaxea fascicularis* (Scleractinia order, Euphylliidae family; Linnaeus, 1758) [47], to explore its suitability for studying the role of oxidative stress in coral bleaching.

Thus, this study investigated the relative roles of ROS and RNS in the thermal stress responses of the sea anemone *E. diaphana* [48] and the coral *Galaxea fascicularis* [47], both from the GBR. We measured a range of physiological traits involved in cnidarian holobiont physiological health during simulated summer heatwaves. We then compared these to the net production of ROS and RNS, as well as to activities of key enzymes in both host and symbiodiniacean components that are involved in ROS scavenging and RNS synthesis. 

## 2. Materials and Methods

### 2.1. Identification of Symbiodiniacean Communities from Galaxea fascicularis Colonies

The *Exaiptasia diaphana* anemones used in our study are known to host *Breviolum minutum* [49]. We also identified the strains of symbiodiniaceans associated with *Galaxea fascicularis* colonies used in this study. Before the start of the experiment, two 1 cm pieces were cut off of one polyp from each coral colony and snap-frozen in liquid nitrogen. DNA of these pieces was extracted separately using previously established protocols [50], with the modification of bead beating the samples with 30 mg sterile glass beads three successive times at 20 s at 30 Hz. We included one negative control for the DNA extraction and two for the subsequent PCRs. Extracted DNA and negative controls were amplified in triplicates using symbiodiniacean ITS2 primers Sym_Var_5.8S2 (5′ GTGACCTATGAACTCAGGAGTCGAATTGCAGAACTCCGTGAACC 3′] [51]; Sym_Var_Rev [3′ CTGAGACTTGCACATCGCAGCCGGGTTCWCTTGTYTGACTTCATGC 5′) [52] according to previously established protocols [46], with the adjustment of using QIAGEN Multiplex PCR Master Mix (QIAGEN, Clayton, VIC, Australia) for the overhang PCRs. Libraries were sequenced at the Walter and Eliza Hall Institute (WEHI, The University of Melbourne) on an Illumina MiSeq V3 system with 2 × 300 bp paired-end reads. For the analysis of symbiodiniacean communities, raw sequences were submitted to SymPortal [53].

### 2.2. Experimental Setups and Sample Processing

**Figure 2:**
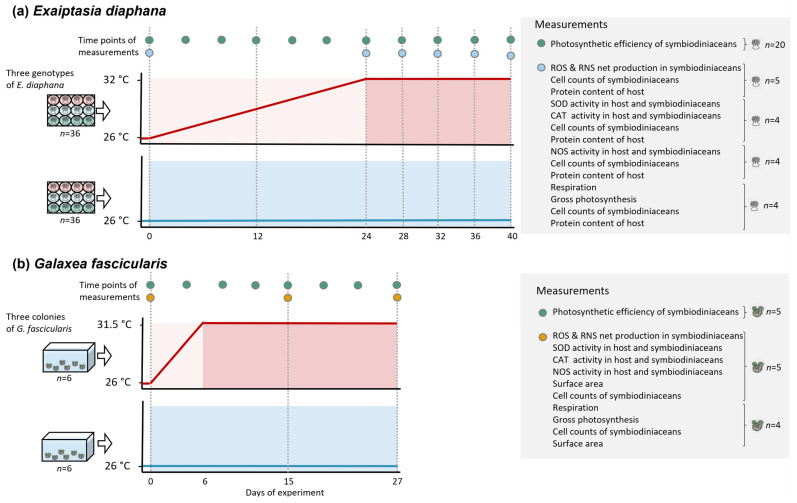
Temperature profiles and sampling schedule of experiments examining the role of ROS and RNS during bleaching in (**a**) *Exaiptasia diaphana* and (**b**) *Galaxea fascicularis*. Experiments were conducted separately for *E. diaphana* and *G. fascicularis* with three genotypes or colonies, respectively, and 36-well plates or 6 tanks per treatment, respectively. Circles represent measurement and sampling timepoints. Parentheses indicate the measurements that were taken with the same specimen. Replicate numbers are given per genotype or colony per temperature treatment on each sampling time point. In *E. diaphana*, each replicate was a single sea anemone, and in *G. fascicularis*, each replicate was a fragment of three polyps.

*Exaiptasia diaphana* sea anemone genotypes AIMS2, AIMS3, and AIMS4, which originated from the Great Barrier Reef (GBR) [54], were selected from the culture collection at the University of Melbourne, Australia (*n* = 288 per genotype) in February 2020. Sea anemones were transferred into one well each of sterile 12-well culture plates (Corning^®^ Costar^®^, CLS3512, Merck, Macquarie Park, NSW, Australia). Each of the total 72 culture plates contained four sea anemones per genotype. Culture plates were equally distributed among four experimental incubators (two incubators per temperature treatment; Model No. LE-509, Thermoline Scientific, Wetherill Park, NSW, Australia) that were equipped with white light-emitting diodes (15–20 μmol photons m^−2^ s^−1^) set to a 12 h light and 12 h dark cycle. The GBR *E. diaphana* is highly sensitive to light, even under ambient temperature. Though we attempted to acclimate the sea anemones over weeks to slightly higher light levels, which were previously applied to these genotypes in our laboratory (28 [46] (Supplemental Table S1) and 31.8–33.8 μmol photons m^−2^ s^−1^ [50] (Supplemental Table S1), the corals showed high mortality under light levels above 20 μmol photons m^−2^ s^−1^. These findings are congruent with another recent study from our laboratory, which consequently used the same low light intensities as in this study [45] (Supplemental Table S1). Sea anemones were kept in 5 mL reconstituted Red Sea Salt^TM^ seawater (RSS, R11065, Red Sea, Houston, TX, USA) at a salinity of 34 ppt, which allowed us to provide the best water quality conditions while being able to maintain large numbers of individuals. A previous study from our laboratory [50] showed that *E. diaphana* can be grown in 5 mL culture wells with no effects on fitness and survival. RSS of each well was changed completely every two days. Sea anemones were fed every four days with freshly hatched *Artemia salina* (Salt Creek, Premium, GSL, Salt Lake City, UT, USA), and wells were cleaned 1–2 h after feeding by removing debris using sterile plastic pipettes and cotton buds, followed by an RSS change. Sea anemones in the ambient treatment were kept at 26 °C for all of the 40 days of the experiment (Figure 2a). In the elevated temperature treatment, sea anemones were subjected to an increase of 0.25 °C per day over 24 days (day 0–24) from 26 °C to 32 °C, followed by constant temperature conditions of 32 °C for 17 days (day 24–40). This temperature treatment was chosen because in a previous study on GBR-sourced *E. diaphana*, the sea anemones bleached at 31.5 °C [46] (Supplemental Table S1); note that we exposed the sea anemones for five more days of constant heat. Samples to assess photosynthesis, respiration (*n* = 12 sea anemones in total per temperature treatment per sampling day, 4 per genotype), net ROS and RNS production (*n* = 15 total, 5 per genotype), symbiodiniacean cell density, and host protein of all fragments were collected on days 0, 13, 24, 28, 32, 36, and 40 of the experiment. Activities of enzymes involved in ROS scavenging and RNS synthesis (*n* = 12 total, 4 per genotype) and their respective symbiodiniacean cell density and host protein were measured on the same days except for day 13. Symbiodiniacean photosynthetic efficiency (*n* = 60 total, 20 per genotype) was determined on days 0, 4, 8, 13, 16, 20, 24, 28, 32, 36, and 40. For assessments of symbiodiniacean cell counts, host protein content, and ROS and RNS production, sea anemones were homogenized individually in 1000 μL 0.22 μm filtered RSS (fRSS) using sterile glass homogenizers. For enzyme activity measurements of SOD and CAT, sea anemones were homogenized on ice in 1000 μL ice-cold PBS 1X. Sea anemones for NOS activity measurements were homogenized on ice in 1000 μL ice-cold 1X protease inhibitor (cOmplete^TM^, Roche, Merck, Macquarie Park, NSW, Australia) in fRSS. Individual methods are detailed below. 

Three colonies of *Galaxea fascicularis* were collected at Davies Reef (Great Barrier Reef, Australia, 3–5 m depth, permit no. G12/35236.1) in August 2021 and brought to the National Sea Simulator at the Australian Institute for Marine Science, Townsville, QLD, Australia. Corals were shipped to the University of Melbourne immediately, where they were kept in a recirculating system containing 120 L of RSS at 35 ppt and light intensities of 90 μmol photons m^−2^ s^−1^ (ZP3600, Zetlight, Hong Kong, SAR of China) until the start of the experiment in November 2021. Three weeks before the start of the experiment, coral colonies were cut into fragments of three polyps (*n* = 144 in total, 48 fragments per colony) using a diamond band saw and glued onto ceramic plugs. Four coral fragments per colony were randomly moved to one of 12 experimental tanks (*n* = 6 tanks per temperature treatment) containing 12 L of RSS at 35 ppt and a wave pump (301F, Aqua One, Morningside, QLD, Australia). Coral fragments could not be maintained in well plates (as was done for *E. diaphana*), because they showed high mortality under these culture conditions. The daily light cycle in each tank was as follows: starting at 7 am, 1.5 h at 10 μmol photons m^−2^ s^−1^ (ZP3600, Zetlight, Hong Kong, SAR of China), 2 h at 50 μmol photons m^−2^ s^−1^, 5 h at 80 μmol photons m−^2^ s−^1^, 2 h at 40 μmol photons m^−2^ s^−1^, 1.5 h at 10 μmol photons m^−2^ s^−1^, and 12 h with no light. Tanks were kept inside a temperature-controlled room, creating an ambient water temperature of 26 ± 1.1 °C. Due to differences in bleaching susceptibility between *G. fascicularis* and *E. diaphana*, the temperature profile was slightly altered for the former (Figure 2b). The elevated temperature treatment for *G. fascicularis* consisted of an increase of 1 °C per day over 5 days, followed by an increase on 1 day of 0.5 °C (day 0–6), and by constant temperature conditions of 31.5 °C for 22 days (day 6–28). The ambient control remained at constant 26 °C for 28 days. Temperature ramping inside of the tanks was performed using aquarium heaters (EHEIM, Deizisau, Germany), and temperatures in each tank were monitored using data loggers (Hobo UA-001-08, OneTemp). Half water changes of experimental tanks were performed every four days, and tanks were cleaned every eight days using scourers. Corals were fed with microalgae (Shellfish Diet 1800^®^, Reed Mariculture, Campbell, CA, USA) every eight days. Samples for determining net ROS and RNS production (*n* = 15 coral fragments in total per treatment per sampling day, 5 per colony), enzyme activities (*n* = 12 total, 4 per colony), and symbiodiniacean cell counts and coral surface area of each fragment were taken on day 0, 15, and 27. Respiration and photosynthesis rates (*n* = 12 total, 4 per colony), as well as symbiodiniacean cell counts and surface area, were assessed on day 0, 16, and 28. Photosynthetic efficiency of coral fragments was measured on day 0, 4, 8, 12, 15, 17, 19, 21, 23, 25, and 27. To obtain samples for measurements of symbiodiniacean cell counts, ROS and RNS production and enzyme activities, tissues of individual polyps were removed using a Waterpik and 50 mL of fRSS. Tissue homogenates were centrifuged at 3000× *g* for 15 min. If samples were collected for enzyme activity measurements, homogenates were kept for analysis of the host compartment. The remaining symbiodiniacean pellet was resuspended in 2000 µL fRSS and homogenized using a glass homogenizer. 

Sampled coral and sea anemone homogenates were used immediately for the detection of ROS and RNS levels, as well as enzyme activities, but were stored at −20 °C for symbiodiniacean cell count measurements and protein content quantification. Coral polyps were bleached, and remaining dead skeletons were kept for surface area measurements.

### 2.3. Photosynthetic Performance and Respiration

Photosynthetic efficiency, measured as the maximum quantum yield (*F*_v_*/F*_m_) of PSII, was taken of *in hospite* symbiodiniaceans in *Exaiptasia diaphana* and *Galaxea fascicularis* using an imaging-PAM fluorometer (IMAG-MAX/L, Heinz Waltz GmbH, Effeltricht, Germany). For *E. diaphana* and *G. fascicularis*, measurements were taken 1–2 h after the start of the light cycle and a dark acclimation of 30 min. PAM settings for *E. diaphana* were: saturating pulse intensity 10, measuring light intensity 4 (frequency 1), damping 2, and gain 4 to achieve a F0 of 0.1–0.3. PAM settings for *G. fascicularis* were: saturating pulse intensity 10, measuring light intensity 2 (frequency 1), damping 2, and gain 2 to accomplish a F0 of 0.1–0.3 as well. 

Measurements of oxygen (O_2_) production and utilization were taken during light and dark incubations of corals and sea anemones to determine net photosynthesis and respiration rates, respectively. Individual sea anemones and coral fragments were put into customized 15 mL and 30 mL transparent plastic vials filled with RSS at 34 and 35 ppt, respectively. Vials were transferred into a temperature-controlled incubator (Model No. LE-509, Thermoline Scientific, Wetherill Park, NSW, Australia) fitted with white light-emitting diodes (20 μmol photons m^−2^ s^−1^) for sea anemones or an aquarium light for coral fragments (ZA2441, Zetlight, Hong Kong SAR; 80 μmol photons m^−2^ s^−1^). Temperatures inside the incubator reflected respective treatment temperatures of specimens, and RSS for filling up the vials was preheated to treatment temperatures. Vials were placed on magnetic stirrer spots inside of the incubator to enable constant mixing of water. Sea anemones and corals were kept in the dark for 30 min inside the incubator for acclimation and temperatures to stabilize. O_2_ concentrations inside of the vials were subsequently measured every 2 s for 1 h in the darkness, followed by 1 h in light conditions (see above). O_2_ was measured using FireSting O_2_ optical oxygen sensors (PyroScience, Aachen, Germany) connected to each vial. Two RSS controls were included in a run of six specimens each. 

To estimate O_2_ fluxes, we fitted linear regressions for each specimen per dark and light incubation using the function *lm* in R 1.4.17 [55]. We corrected O_2_ fluxes for RSS controls and normalized them to sea anemone host protein or coral surface area (see below). Gross photosynthesis was estimated as the sum of net photosynthesis and respiration rates.

### 2.4. Symbiodiniacean Cell Density, Sea Anemone Host Protein, and Coral Surface Area 

Stored homogenates of *Exaiptasia diaphana* were thawed and centrifuged at 2300× *g* for 5 min to pellet symbiodiniaceans. The supernatant was saved for host protein analysis. Pelleted symbiodiniaceans were resuspended in 1 mL fRSS, centrifuged, and resuspended in 1 mL fRSS again. Further measurements of symbiodiniacean cell counts and protein contents were conducted as previously described [45], using, respectively, an automated cell counter (Countess II FL, Thermo Fisher Scientific, Scoresby, VIC, Australia) and a Bradford assay. Symbiodiniacean cell counts were normalized to host protein contents.

Preserved homogenates of *Galaxea fascicularis* were centrifuged at 2300 × *g*, discarding the supernatant, and resuspended in 2 mL fRSS. After performing this twice, 300 µL of the resuspended symbiodiniacean pellet was filtered through a 40 µm cell strainer (pluriSelect, Leipzig, Germany) and vortexed, followed by measurements with a CytoFLEX LX flow cytometer (Beckman Coulter, Brea, CA, USA). Singlet symbiodiniacean cells were gated and counted as described in 2.5. We estimated symbiodiniacean cell counts from *G. fascicularis* samples using a flow cytometer instead of an automated cell counter as for *E. diaphana*, as *G. fascicularis* tissues exhibited high autofluorescence. Thus, higher auto fluorescent content was counted as symbiodiniacean cells by the automated cell counter, whereas flow cytometry could discriminate between coral autofluorescence and fluorescence by symbiodiniaceans.

Coral surface area was estimated using 3D computer modeling [56]. Here, we took ~90 photos of each bleached coral fragment from nine horizontal and five vertical angles and two different distances. Fragments were placed on a stand with six attached markers (provided by Agisoft Metashape Professional 1.8) and a blue background. The photos were used to create 3D models of each fragment and to estimate its surface area after closing holes of the created model in Agisoft Metashape Professional 1.8.

### 2.5. ROS and RNS Quantification in Freshly Isolated Symbionidiaceae by Flow Cytometry

Net production of either ROS or RNS is defined here as the difference between the amount produced and the amount neutralized within the coral or sea anemone holobiont. Net ROS levels were quantified with CellROX^®^ Orange (Thermo Fisher Scientific, Scoresby, VIC, Australia), as previously described [45]. CellROX^®^ Orange is widely used to detect general ROS but has been found to be more sensitive to H_2_O_2_ than O_2_^−^ [57]. A volume of 3 µL of CellROX^®^ Orange (stock at 500 µM in dimethyl sulfoxide (DMSO); final concentration 5 µM) was added to 300 µL of either sea anemone or coral homogenate. Samples were incubated at sample experimental light levels and temperatures for 90 min and processed within 60 min to maximize dye stability. Net RNS levels were quantified with DAF-FM DA (4-Amino-5-Methylamino-2′,7′-Difluorofluorescein Diacetate) (Thermo Fisher Scientific, Scoresby, VIC, Australia), which detects nitric oxide [36,37,39]. A volume of 15 µL of DAF-FM DA (stock at 500 µM in DMSO; final concentration 15 µM) was added to 300 µL of either sea anemone or coral homogenate. Samples were incubated in the dark for 30 min at sample experimental temperatures. Samples were then washed by centrifugating at 2300× *g*, discarding the supernatant, and resuspending in 1 mL fRSS (*Exaiptasia diaphana*) or 2 mL fRSS (*Galaxea fascicularis*). This was performed twice, and 300 µL fRSS of the final samples were taken. As a control, 3 µL of DMSO was added to the remaining 300 µL of either sea anemone or coral homogenate, which were incubated at sample experimental light levels and temperatures for 90 min. 

Samples were filtered through a 40 µm cell strainer (pluriSelect, Leipzig, Germany) and vortexed before processing with a CytoFLEX LX flow cytometer (Beckman Coulter, Brea, CA, USA) at a speed of 20 µL/min. The same gating procedure was followed for both sea anemone and coral samples (Supplemental Figure S1). Autofluorescent events strongly excited by the 405 nm laser at emissions 450 ± 22.5 nm and 763 ± 21.5 nm were gated to separate symbiodiniaceans from host debris and bacteria. An FSC-A/SSC-A plot was then used to select homogenous cells, and singlets were gated on an FSC-H/FSC-A plot. For each sample, the CellROX^®^ Orange signal was quantified as the median signal of stained minus unstained cells on single cells gated on previous plots with the 561 nm laser (emission 585 ± 21 nm). The DAF-FM DA signal (RNS dye) was quantified as the median signal of stained minus unstained cells on single cells gated on previous plots with the 488 nm laser (emission 525 ± 20 nm). At least 10,000 symbiodiniacean singlets per sample were processed, except in samples in which symbiodiniacean density was low because of advanced bleaching.

Prior to RNS quantification and to confirm the flow cytometry results, 200 µL of DAF-FM-DA-stained and unstained samples used for flow cytometry were pipetted onto poly-L-lysine (0.1% *w*/*v* in Milli-Q water)-coated 8-well coverslip-bottom well slides (ibidi, Gräfelfing, Germany). DAF-FM DA signal in symbiodiniacean cells was then visualized with an inverted confocal laser scanning microscope (Nikon A1R, Nikon, Melville, NY, USA) by excitation at 488 nm (emission 497–557 nm), and symbiodiniacean cells were visualized through their chlorophyll fluorescence by excitation at 488 nm (excitation 600–710 nm; Supplemental Figure S2). Images were processed with ImageJ. CellROX^®^ signal was previously observed to stain the symbiodiniacean cell wall and symbiosome lumen [45], and no further observations were performed in this study.

### 2.6. Enzyme Activities Involved in ROS Scavenging and RNS Synthesis

A volume of 500 µL of freshly collected homogenates of *Exaiptasia diaphana* and *Galaxea fascicularis* in PBS (for SOD and CAT measurements) or in 1X protease inhibitor in fRSS (NOS measuremens) were centrifuged at 5000× *g* for 10 min at 4 °C to pellet symbiodiniaceans. Supernatants were collected for enzyme analysis of the host compartment and kept on ice. Pelleted symbiodiniaceans were resuspended in 500 µL ice-cold PBS or 1X protease inhibitor in fRSS. For normalization of enzyme activities, aliquots of each symbiodiniacean and host compartment were stored in −20 °C for subsequent symbiodiniacean cell counts (*E. diaphana* and *G. fascicularis*) and host protein analysis (*E. diaphana*), as described in 2.4.

To detect SOD total activities (i.e., Cu/Zn, Mn, and Fe SOD), we used a colorimetric determination kit (EIASODC, Thermo Fisher Scientific, Scoresby, VIC, Australia). Before conducting the assay according to the manufacturer’s protocol, 100 µL of each symbiodiniacean and host homogenate was diluted with 100 µL of the provided assay buffer to obtain values within the range of the supplied SOD standards. Absorbance was measured at 450 nm (CLARIOstar PLUS plate reader, BMG Labtech, Mornington, VIC, Australia) at room temperature against SOD standards. Values are expressed in the standard cytochrome c SOD unit (U mL^−1^). 

CAT activities were assessed similarly as previously described [58]. Here, we added 20 µL of either symbiodiniacean or host compartment of each sea anemone or scleractinian homogenate to wells of a 96-well plate, and 30 μL of PBS and 50 μL of 50 mM H_2_O_2_ were added to each well. Blanks containing 20 µL PBS instead of samples were included. We measured the absorbance at 240 nm immediately and after 10 min (CLARIOstar PLUS plate reader, BMG Labtech, Mornington, VIC, Australia). CAT activity is expressed here as mM H_2_O_2_ scavenged (initial absorbance–final absorbance) per minute.

NOS activities were measured using a colorimetric assay kit (ab211083, abcam, Melbourne, VIC, Australia), following the manufacturer’s protocol and using 60 μL of sample to achieve values within the given NOS standards. Absorbance was measured at 540 nm against standards, and values are expressed in NOS specific activity (mU mL^−1^).

SOD, CAT, and NOS activities of symbiodiniacean compartments were normalized to symbiodiniacean cell counts per mL, and host compartments were normalized to host protein (*E. diaphana*) or surface area (*G. fascicularis*).

### 2.7. Data Analysis

All datasets were analyzed using R v1.4.17 [55]. We merged all collected data from each sea anemone genotype or coral colony per temperature treatments, which increased replication by a factor of three. After testing for normal distribution using a Shapiro–Wilk test, measured parameters were analyzed using generalized linear mixed effect models (glm function, GLM) by incorporating the temperature treatment as well as the sampling time point as fixed factors. Pairwise comparisons based on created GLMs were conducted via Tukey HSD post hoc tests, by applying the function *lsmeans* in R. Results of each measured parameter per temperature treatment and cnidarian holobiont were plotted in R using the package “ggplot2” v3.3.5 [59].

## 3. Results

### 3.1. Bleaching Metrics of Exaiptasia diaphana and Galaxea fascicularis

**Figure 3:**
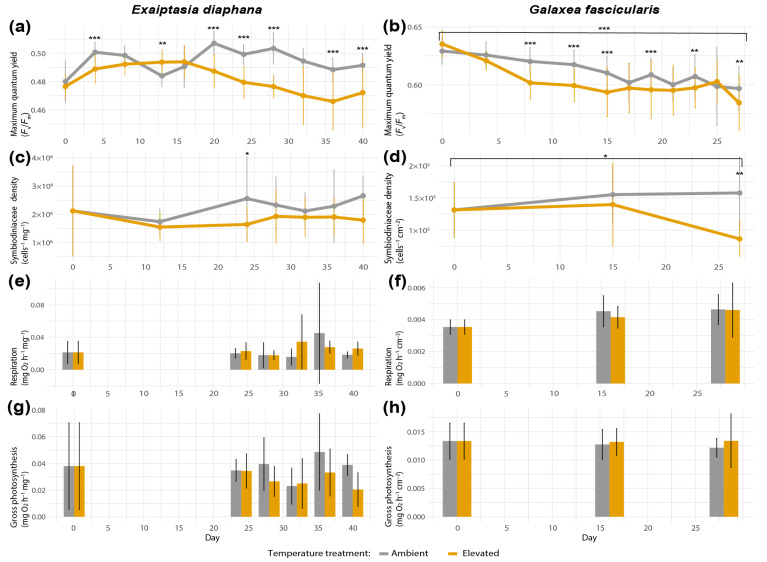
Bleaching metrics of *Exaiptasia diaphana* and *Galaxea fascicularis*, each comprising (**a**,**b**) maximum quantum yield of symbiodiniaceans (*F*_v_*/F*_m_) (*n* = 60 per treatment), (**c**,**d**) symbiodiniacean density (*n* = 15), (**e**,**f**) holobiont respiration rates (*n* = 12), and (**g**,**h**) gross photosynthesis rates (*n* = 12). Bars depict standard deviation. Data shown for day 0 for both temperature treatments correspond to the same replicates. Asterisks display statistical significance according to Tukey’s HSD post hoc pairwise comparisons based on generalized linear models (*p*-value < 0.001 = ***, <0.01 = **, <0.05 = *).

Maximum quantum yield (*F*_v_*/F*_m_) in *Exaiptasia diaphana* differed significantly between values of control versus heat-exposed individuals on day 4 (Tukey’s post hoc pairwise comparisons based on generalized linear models (GLM), *p* < 0.0001, Figure 3a), day 14 (GLM, Tukey’s post hoc test, *p* = 0.0039), and days 20, 24, 28, 32, 37, and 40 (GLM, Tukey’s post hoc test, *p* < 0.0001 for all listed timepoints). However, *F*_v_*/F*_m_ values in heat-stressed *E. diaphana* did not differ significantly between the first and last day of the experiment (day 0 versus 40, GLM, Tukey’s post hoc test, *p* = 0.93, Figure 3a). Furthermore, symbiodiniacean densities in heat-treated *E. diaphana* did not decrease significantly over time (day 0 versus 40, GLM, Tukey’s post hoc test, *p* = 0.97, Figure 3c) and were only significantly different from control sea anemones on day 24 (GLM, Tukey’s post hoc test, *p* = 0.0180; Figure 3c). In *Galaxea fascicularis*, a significant decrease in maximum quantum yield of symbiodiniaceans in the elevated treatment between the end and start of the study (day 0 versus 27, GLM, Tukey’s post hoc test, *p* < 0.0001, Figure 3b) was observed. Although yields also decreased in the control corals over time, at most time points, *F*_v_*/F*_m_ values were significantly higher in the control compared with heat-exposed corals (GLM, Tukey’s post hoc test, days 8, 12, 15, 19: *p* < 0.0001, day 23: *p* = 0.0046, day 27: *p* = 0.0021, Figure 3b). Additionally, *G. fascicularis* exhibited a significant difference in symbiodiniacean densities between elevated and control treatments on the last day of the experiment (reduced by 0.45-fold, day 27, GLM, Tukey’s post hoc test, *p* = 0.002, Figure 3d), as well as a significant decrease in symbiodiniacean densities in the elevated treatment between the first and last day of the experiment (reduced by 0.47-fold, day 0 versus 27, GLM, Tukey’s post hoc test, *p* = 0.041). Respiration and gross photosynthesis rates did not differ significantly between temperature treatments across time in either holobionts (Figure 3e–h), but variability within and between sea anemone genotypes and coral colonies was very high, and larger sample sizes may have been required to detect possible differences (Supplemental Figures S5 and S6). The three colonies of *G. fascicularis* were exclusively associated with *Durusdinium trenchii* (ITS2 profile D1-D4-D4c-D1ev-D10, Supplemental Table S2).

### 3.2. Net ROS and RNS Production during Thermal Stress in Exaiptasia diaphana and Galaxea fascicularis

**Figure 4:**
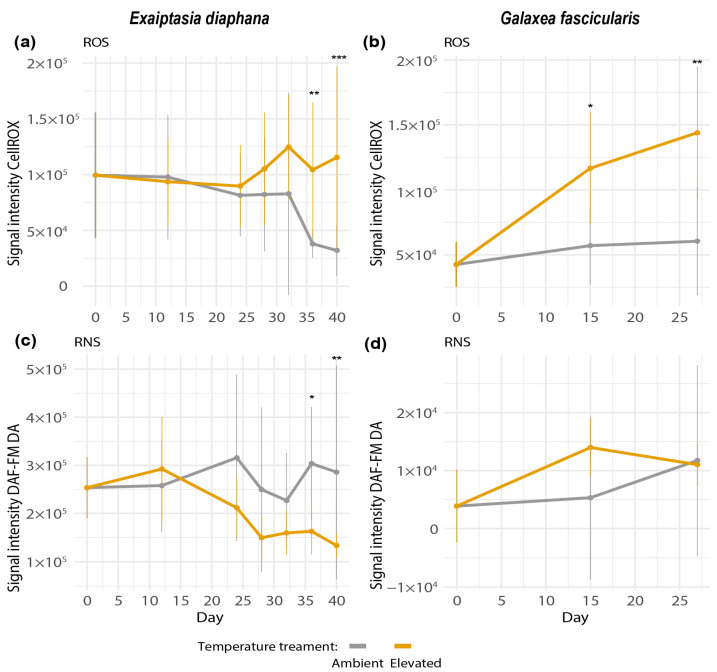
Net ROS production of (**a**) *Exaiptasia diaphana* and (**b**) *Galaxea fascicularis* and net RNS production of (**c**) *E. diaphana* and (**d**) *G. fascicularis*. Net ROS was measured as signal intensity of CellROX-stained symbiodiniacean cells. Net RNS was measured as signal intensity of DAF-FM DA-stained symbiodiniacean cells. Signal intensities were normalized by subtracting the median signal of unstained cells to control for autofluorescence. Bars depict standard deviation. *N* = 15 per temperature treatment. Data shown for day 0 for both temperature treatments correspond to the same replicates. Asterisks display statistical significance according to Tukey’s HSD post hoc pairwise comparisons based on generalized linear models (*p*-value < 0.001 = ***, <0.01 = **, <0.05 = *).

In both holobionts, net ROS production increased during thermal stress and was higher in heat-exposed than control samples at the last two sampling time points (GLM, Tukey’s post hoc test, *Exaiptasia diaphana* day 36: *p* = 0.008, day 40: *p* = 0.007; *Galaxea fascicularis* day 15: *p* = 0.0140, day 27: *p*= 0.0024, Figure 4a,b). However, net ROS production in elevated temperature treatments between the start and end of the experiments was only significantly different in *G. fascicularis* (day 0 versus 27, GLM, Tukey’s post hoc test, *p* = 0.0001, Figure 4b). The significant difference in net ROS between elevated and ambient temperatures in *E. diaphana* at days 36 and 40 might have only emerged because of a decrease in net ROS under ambient temperatures towards the end of the experiment (Figure 4a). The observed increase in net ROS in *E. diaphana* is less biologically relevant, as large variations between genotypes were observed (Supplemental Figure S7a). In comparison, the observed significant increase in net ROS in the elevated temperature treatment was consistent across the three colonies of *G. fascicularis* (Supplemental Figure S7b).

Net RNS production did not change in heat-treated holobionts during the temperature ramping up (Figure 4c,d). A statistically significant decrease in net RNS in the elevated treatment was observed for *E. diaphana* on the last two sampling time points of the experiment (GLM, Tukey’s post hoc test, day 37: *p* = 0.015, day 40: *p* = 0.001, Figure 4c). 

### 3.3. Activities of ROS-Scavenging and RNS-Synthesizing Enzymes during Thermal Stress in Exaiptasia diaphana and Galaxea fascicularis

**Figure 5:**
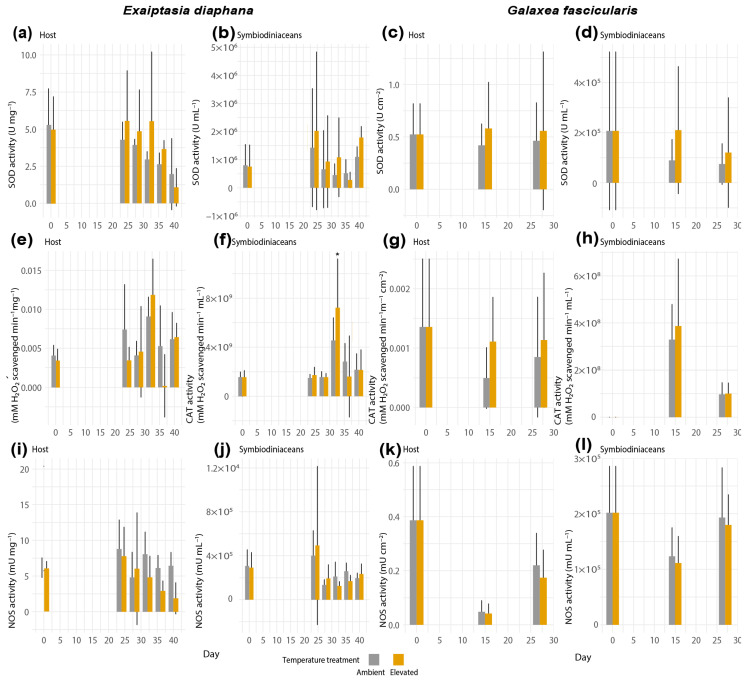
Activities of SOD in (**a**) host and (**b**) symbiodiniacean compartments of *Exaiptasia diaphana*, and in (**c**) host and (**d**) symbiodiniacean compartments of *Galaxea fascicularis*. Activities of CAT in (**e**) host and (**f**) symbiodiniacean compartments of *E. diaphana*, and in (**g**) host and (**h**) symbiodiniacean compartments of *G. fascicularis*. Activities of NOS in (**i**) host and (**j**) symbiodiniacean compartments of *E. diaphana*, and in (**k**) host and (**l**) symbiodiniacean compartments of *G. fascicularis*. Bars depict standard deviation. *N* = 12 per temperature treatment. Data shown for day 0 for both temperature treatments correspond to the same replicates. Asterisks display statistical significance according to Tukey’s HSD post hoc pairwise comparisons based on generalized linear models (*p*-value < 0.05 = *).

Antioxidant enzyme activities did not show statistically significant results between control and heat-exposed samples of any holobionts (Figure 5), except for a significant increase in CAT activity of the symbiodiniacean compartment from heat-stressed *Exaiptasia diaphana* on day 32 (GLM, Tukey’s post hoc test, *p* = 0.027, Figure 5f). The lack of statistical significance in the remaining response variables might stem from high variability between and within sea anemone genotypes and coral colonies, respectively (Supplemental Figures S8 and S9). 

Activities of the RNS-synthesizing enzyme NOS in both compartments of *E. diaphana* and *Galaxea fascicularis* remained similar between ambient and elevated treatments throughout the experiment (Figure 5i–l). 

## 4. Discussion

To study the relative roles of ROS and RNS in the cnidarian heat-stress response, we compared the relative production of ROS and RNS in symbiodiniacean cells and activities of key enzymes involved in their synthesis or degradation in host and symbiodiniacean compartments of *Exaiptasia diaphana* and *Galaxea fascicularis*. Our results suggest that these oxygen radicals play a greater role in the heat stress response compared to the nitrogen radicals, especially in *G. fascicularis*. 

### 4.1. GBR-Sourced Exaiptasia diaphana Is Not a Suitable Model to Study Coral Bleaching

Differences in bleaching metrics between elevated and ambient treatments indicated thermal bleaching occurred in *Galaxea fascicularis* but not in *Exaiptasia diaphana*. *Exaiptasia diaphana* has been an important model in the coral field for studying the establishment and maintenance of the cnidarian-symbiodiniacean symbiosis. However, there seem to be distinct differences in the breakdown of this symbiosis between *G. fascicularis* and *E. diaphana* [54], which may be due to a number of factors, including the holobionts harboring different communities of symbiodiniaceans (*Durusdinium trenchii* in *G. fascicularis* and *Breviolum minutum* in *E. diaphana* [49]).

Unexpectedly, the bleaching metrics of *E. diaphana* observed here differ from those observed in other studies from our laboratory that used the same GBR-sourced genotypes [45,46,50] (Supplemental Table S1). Specifically, one study that applied similar elevated temperatures and duration of heat exposure and used the same three *E. diaphana* genotypes showed significant loss of symbiodiniacean cells [46] (Supplemental Table S1), although not in all genotypes. It is possible that these inconsistent results reflect the facultative nature of the symbiodiniacean-*E. diaphana* symbiosis [60,61]. Additionally, other laboratories using *E. diaphana* from different geographic locations have been able to expose them to higher light intensities, such as 40 [62], 70 [44], or 95 [63] photons m^−2^ s^−1^. The lower light levels we were limited to using in the present *E. diaphana* experiment (15–20 photons m^−2^ s^−1^) may contribute to the observed differences in bleaching severity between the two holobionts, and the high light sensitivity makes GBR *E. diaphana* an inappropriate model to study coral bleaching. GBR-sourced *E. diaphana* remains a suitable model to study other aspects of the cnidarian-symbiodiniacean symbiosis [45], but we recommend using the emerging model, *G. fascicularis*, as a model for coral bleaching. This species is hardy, can be easily fragmented [43], and can be maintained in basic aquarium facilities.

### 4.2. The Oxidative Stress Response during Thermal Stress Was More Marked in Galaxea fascicularis than in Exaiptasia diaphana and Aligned with the Extent of Physiological Stress

**Figure 6:**
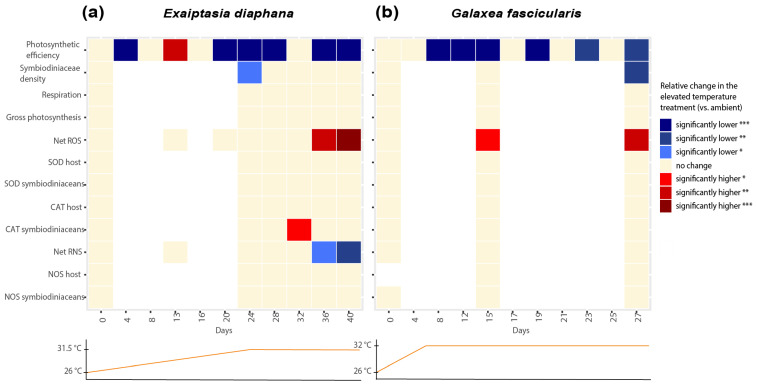
Heatmap showing relative changes in measured parameters in the elevated compared to the ambient temperature treatments over the course of the experiments on (**a**) *Exaiptasia diaphana* and (**b**) *Galaxea fascicularis*. Asterisks display statistical significance according to Tukey’s HSD post hoc pairwise comparisons based on generalized linear models (*p*-value < 0.001 = ***, <0.01 = **, <0.05 = *). Graphs below each heatmap show the temperature profile of the elevated treatment for each experiment.

The measurements of net ROS and RNS and relevant enzymes suggest that ROS production may play a role in thermal stress response in both *Exaiptasia diaphana* and *Galaxea fascicularis* (Figure 6). 

Even though no bleaching was observed in *E. diaphana*, we still observed moderately higher levels of ROS and increased activity of an antioxidant enzyme, indicative of oxidative stress. The thermal stress response in *E. diaphana* (day 32) was characterized by significantly higher CAT activities in symbiodiniacean cells (day 32, Figure 6a). This suggests that H_2_O_2_ concentrations may have increased in the symbionts, as indicated by increases in CAT activity (CAT scavenges H_2_O_2_). Subsequently, net ROS in symbiodiniacean cells from *E. diaphana* increased moderately at elevated temperatures compared to the control in the last days of the experiment (day 36 and 40). This increase in ROS was moderate, because the difference in ROS between elevated and ambient temperatures was also driven by a decrease in the ambient treatment. Our results partially resemble previously described oxidative stress cascades in corals, i.e., the increase in CAT activities at the beginning of thermal stress [5,7]. However, it remains unclear to what extent ROS played a role during thermal stress in GBR *E. diaphana*, since *E. diaphana* only displayed a moderate heat stress response in this study (which, as mentioned earlier, may have been due to the low light levels required). Nonetheless, a recent study that applied the same light levels observed significant signs of thermal bleaching in GBR *E. diaphana* but demonstrated no increases in ROS, postulating no evidence for the oxidative stress theory of bleaching in these GBR sea anemones [45]. 

In *G. fascicularis*, both physiological measures of stress and the cascade of antioxidant responses were more marked than in *E. diaphana*. We measured significant increases of net ROS not only between elevated and ambient temperatures at both later time points (day 15 and 27), but also between the start and end of the experiment. This is consistent with the significant physiological signs of bleaching observed in *G. fascicularis*. At the beginning of the thermal stress experiment in *G. fascicularis* (day 15, Figure 6b), net ROS in symbiodiniacean cells already increased significantly in elevated compared to ambient treatments. By the time symbiodiniacean cell densities had declined significantly in heat-stressed *G. fascicularis* (day 27), net ROS production in symbiodiniacean cells had increased even further. The lack of differences in the activities of CAT and SOD between treatments throughout the sampling time points suggests that enzymes were already overwhelmed by the high ROS levels on day 15 and may have been inactivated. Indeed, increased concentrations of O_2_^−^ (from 1 nM) inactivate enzymes [64], and high concentrations of H_2_O_2_ (0.01 to 2 M) reversibly inhibit and irreversibly inactivate mammalian CAT [65]. The ROS levels by which CAT and SOD are inactivated in corals remain to be investigated and may depend on a range of factors, such as the degree of adaptation of the holobiont to thermal stress or the taxonomic identity of the symbiodiniacean and/or coral host species. For corals, O_2_^−^ production rates have only been reported once [66], and H_2_O_2_ has been reported twice [67,68]. Thus, the levels of H_2_O_2_ and O_2_^−^ during bleaching in most coral species are currently unknown, as are the levels that might cause inactivation of antioxidant enzymes. In contrast, a previous study has shown significant trends of increased CAT and SOD activities in *G. fascicularis* host cells and symbiodiniaceans under thermal stress, although net ROS production was not quantified [69]. Since *G. fascicularis* was only exposed to 31 °C for up to 5 days in the previous study [69], it remains unclear whether the longer duration of thermal stress (9 days at constant 31.5 °C on day 15) and the significantly increased levels of ROS measured at day 15 in this study might have contributed to SOD and CAT having already reached a state of overburdening. Measuring antioxidant enzyme activities and ROS production at an earlier timepoint after temperatures reached 31.5 °C may have unraveled this question. Despite these differences, enhanced ROS production during thermal stress in *G. fascicularis* is consistent with the oxidative stress hypothesis of coral bleaching [5,6,7].

### 4.3. Ambiguous Role of RNS in the Thermal Stress Response of Exaiptasia diaphana and Galaxea fascicularis

The role of RNS during thermal stress was much more ambiguous than the role of ROS in both holobionts (Figure 6). Net RNS in symbiodiniacean cells was never significantly higher at elevated compared to ambient temperatures. In fact, net RNS production significantly decreased in heat-stressed *Exaiptasia diaphana*. These findings contrast with previous studies showing significant increases in RNS, measured with the same fluorescent probe, during thermal stress of *E. diaphana* from Florida (United States) [37]. The observed differences may be due to the sample’s genetic identity, different light levels used, or the different symbiodiniacean communities harbored. However, a recent study also found a significant decrease in RNS of thermally stressed symbiodiniaceans (*Breviolum minutum*) [70]. A possible scenario for not being able to detect an increase but a decrease in RNS in this study could be that it may have instantly reacted with OH^−^ to form ONOO^−^. The formation of ONOO^−^ from NO has been described to be part of the bleaching cascade when increased OH^−^ becomes available during thermal stress, but we did not measure this in our model system. ONOO^−^ has been found to increase in symbiodiniaceans from *Aiptasia pulchella* under heat stress [40]. Alternatively, downregulation of RNS production may be a strategy to survive short-term heat exposure, as previously hypothesized [43]. Future studies comparing RNS responses to acute thermal stress between cnidarian holobionts that differ in thermal tolerance could shed more light on this theory.

Our findings for *Galaxea fascicularis* suggest a lesser role of RNS in its thermal stress response compared to ROS. This is a novel observation for *G. fascicularis* and differs from recent findings in the coral *Pocillopora acuta*. Though freshly isolated symbiodiniaceans from thermally stressed *G. fascicularis* showed no increases in RNS but strong increases in ROS production, freshly isolated symbiodiniaceans from thermally stressed *P. acuta* showed higher RNS and minimal ROS production with increasing temperatures [43]. Interestingly, the study found that the pronounced increase in RNS in *P. acuta* was only linked to larger increases in temperature (~+2 °C), and a stronger role of ROS and more extreme physiological stress responses were detected at moderate thermal stress (~+0.5 °C) [43]. Since thermal thresholds of *G. fascicularis* from the GBR have not been studied before, further studies measuring ROS and RNS production in *G. fascicularis* exposed to different temperature regimes may unravel whether the relative roles of ROS and RNS vary with the degree of thermal stress.

## 5. Conclusions

There is serious concern that long-lived corals may not be able to naturally adapt fast enough to rising ocean temperatures, with a recent model predicting that environmental conditions will become unsuitable for coral reefs by 2035 [71]. Though it is necessary to counteract the root causes of climate change, a new field of research has emerged that aims to accelerate coral adaptation to climate change [72]. The concept of assisted evolution in corals includes intra- and inter-specific hybridization of the coral host [73], selective breeding within populations, preconditioning, and manipulation of coral-associated symbionts such as symbiodiniaceans [74,75] and bacteria [46,76,77,78,79]. To develop successful bleaching mitigation methods, it is critical to understand the proximate cellular mechanisms that drive coral bleaching, particularly those of coral models.

Our findings indicate that *Galaxea fascicularis* represents a suitable model to study the role of ROS in thermally induced coral bleaching. This is important, as many approaches for the mitigation of coral bleaching aim to enhance ROS scavenging within cnidarian holobionts by assisted evolution approaches, particularly via bacterial microbiome manipulation [46,78,79,80]. When developing such ROS-scavenging approaches, it is crucial that the role of ROS is well understood in the model used. Bleaching responses and ROS production in thermally stressed *G. fascicularis*, compared to *E. diaphana*, both from the GBR, were more similar to patterns observed in other coral species. As corals of the genus *Galaxea* from the GBR have shown to associate with symbiodiniaceans of the genera *Cladocopium* and/or *Durusdinium* [81,82], and this study tested *G. fascicularis* colonies harboring *Durusdinium* only, it remains to be investigated if and how thermal stress responses are affected by the composition of the associated symbiodiniacean communities. *Galaxea fascicularis* is found in a wide range of reef habitats worldwide and is a dominant species on some inshore fringing reefs [83]. *Galaxea fascicularis* is already an emerging model in the coral field [47], due to the ease by which coral colonies can be fragmented, its ability to thrive in simplified aquarium systems, and the potential to induce the loss of symbiodiniaceans from its tissues by menthol bleaching [47]. This study provides the first detailed insight into ROS and RNS production and scavenging during thermal stress in *G. fascicularis*, which will help establish *G. fascicularis* as a coral model. 

## Data Availability

Raw Illumina MiSeq data are available under NCBI BioProject ID PRJNA954555 (http://www.ncbi.nlm.nih.gov/bioproject/954555, accessed on 3 May 2023). All remaining data generated or analyzed in this study are included in this published article and its Supplementary Materials.

## Supplementary Materials

The following supporting information can be downloaded at: https://www.mdpi.com/article/10.3390/antiox12051057/s1, Figure S1: ROS and RNS quantification in symbiodiniaceans freshly isolated from (a) *Exaiptaisa diaphana* and (b) *Galaxea fascicularis*; Figure S2: Confocal laser scanning micrographs of A: cultured unstained *Breviolium minutum* (MMSF01, isolated from *Exaiptasia diaphana*), B: DAF-FM-DA stained *B. minutum*, C,D: DAF-FM-DA stained *E. diaphana* homogenate; Figure S3: (a) Maximum quantum yield of symbiodiniaceans (*F*_v_*/F*_m_) (*n* = 20 per genotype per treatment) and (b) symbiodiniacean cell density (*n* = 5) of *Exaiptasia diaphana*; Figure S4: (a) Maximum quantum yield of symbiodiniaceans (*F*_v_*/F*_m_) (*n* = 20 per colony per treatment) and (b) symbiodiniacean cell density (*n* = 5) of *Galaxea fascicularis*; Figure S5: (a) Holobiont respiration rates (*n* = 4 per genotype per treatment) and (b) gross photosynthesis rates (*n* = 4) of *Exaiptasia diaphana*; Figure S6: (a) Holobiont respiration rates (*n* = 4 per colony per treatment) and (b) gross photosynthesis rates (*n* = 4) of *Galaxea fascicularis*; Figure S7: Net ROS production of (a) *Exaiptasia diaphana* and (b) *Galaxea fascicularis* and net RNS production of (c) *E. diaphana* and (d) *G. fascicularis*; Figure S8: Activities of SOD in (a) host and (b) symbiodiniaceans, of CAT in (c) host and (d) symbiodiniaceans and of NOS in (e) host and (f) symbiodiniacean compartments of *Exaiptasia diaphana*; Figure S9: Activities of SOD in (a) host and (b) symbiodiniaceans, of CAT in (c) host and (d) symbiodiniaceans and of NOS in (e) host and (f) symbiodiniacean compartments of *Galaxea fascicularis*, Table S1: Comparison of experimental settings and results of bleaching parameters of studies using GBR-sourced *Exaiptasia diaphana* genotypes, Table S2: Symbiodiniacean communities of the three coral colonies used in this study.

## References

[1] Hoegh-Guldberg O. (1999). Climate Change, Coral Bleaching and the Future of the World’s Coral Reefs. Mar. Freshw. Res..

[2] Muscatine L., Cernichiari E. (1969). Assimilation of Photosynthetic Products of Zooxanthellae by a Reef Coral. Biol. Bull..

[3] Grottoli A.G., Rodrigues L.J., Palardy J.E. (2006). Heterotrophic Plasticity and Resilience in Bleached Corals. Nature.

[4] Suggett D.J., Smith D.J. (2020). Coral Bleaching Patterns Are the Outcome of Complex Biological and Environmental Networking. Glob. Chang. Biol..

[5] Oakley C.A., Davy S.K. (2018). Cell Biology of Coral Bleaching. Coral Bleaching.

[6] Weis V.M. (2008). Cellular Mechanisms of Cnidarian Bleaching: Stress Causes the Collapse of Symbiosis. J. Exp. Biol..

[7] Szabó M., Larkum A.W.D., Vass I. (2020). A Review: The Role of Reactive Oxygen Species in Mass Coral Bleaching. Photosynthesis in Algae: Biochemical and Physiological Mechanisms.

[8] Apel K., Hirt H. (2004). Reactive Oxygen Species: Metabolism, Oxidative Stress, and Signal Transduction. Annu. Rev. Plant Biol..

[9] Krieger-Liszkay A. (2005). Singlet Oxygen Production in Photosynthesis. J. Exp. Bot..

[10] Mehler A.H. (1951). Studies on Reactions of Illuminated Chloroplasts. II. Stimulation and Inhibition of the Reaction with Molecular Oxygen. Arch. Biochem. Biophys..

[11] Suggett D.J., Warner M.E., Smith D.J., Davey P., Hennige S., Baker N.R. (2008). Photosynthesis and Production of Hydrogen Peroxide by *Symbiodinium* (Pyrrhophyta) Phylotypes with Different Thermal Tolerances. J. Phycol..

[12] Asada K., Allen J., Foyer C.H., Matthijs H.C.P. (2000). The Water-Water Cycle as Alternative Photon and Electron Sinks. Philos. Trans. R. Soc. B Biol. Sci..

[13] Asada K. (1987). Production and Scavenging of Active Oxygen in Photosynthesis. Photoinhibition.

[14] Halliwell B., Gutteridge J.M.C. (1990). Role of Free Radicals and Catalytic Metal Ions in Human Disease: An Overview. Methods Enzymol..

[15] Lesser M.P., Shick J.M. (1989). Effects of Irradiance and Ultraviolet Radiation on Photoadaptation in the Zooxanthellae of *Aiptasia pallida*: Primary Production, Photoinhibition, and Enzymic Defenses against Oxygen Toxicity. Mar. Biol..

[16] Merle P.L., Sabourault C., Richier S., Allemand D., Furla P. (2007). Catalase Characterization and Implication in Bleaching of a Symbiotic Sea Anemone. Free Radic. Biol. Med..

[17] Roberty S., Fransolet D., Cardol P., Plumier J.C., Franck F. (2015). Imbalance between Oxygen Photoreduction and Antioxidant Capacities in *Symbiodinium* Cells Exposed to Combined Heat and High Light Stress. Coral Reefs.

[18] Matta J.L., Govind N.S., Trench R.K. (1992). Polyclonal Antibodies against Iron-Superoxide Dismutase from *Escherichia coli* B Cross-React with Superoxide Dismutases from *Symbiodinium microadriacticum* (Dinophyceae). J. Phycol..

[19] Shick J.M., Lesser M.P., Dunlap W.C., Stochaj W.R., Chalker B.E., Won J.W. (1995). Depth-Dependent Responses to Solar Ultraviolet Radiation and Oxidative Stress in the Zooxanthellate Coral *Acropora microphthalma*. Mar. Biol..

[20] Richier S., Merle P.L., Furla P., Pigozzi D., Sola F., Allemand D. (2003). Characterization of Superoxide Dismutases in Anoxia- and Hyperoxia-Tolerant Symbiotic Cnidarians. Biochim. Biophys. Acta-Gen. Subj..

[21] Lesser M.P., Farrell J.H. (2004). Exposure to Solar Radiation Increases Damage to Both Host Tissues and Algal Symbionts of Corals during Thermal Stress. Coral Reefs.

[22] Plantivaux A., Furla P., Zoccola D., Garello G., Forcioli D., Richier S., Merle P.L., Tambutté É., Tambutté S., Allemand D. (2004). Molecular Characterization of Two CuZn-Superoxide Dismutases in a Sea Anemone. Free Radic. Biol. Med..

[23] Lesser M.P., Shick J.M. (1989). Photoadaption and Defenses against Oxygen Toxicity in Zooxanthellae from Natural Populations of Symbiotic Cnidarians. J. Exp. Mar. Bio. Ecol..

[24] Krueger T., Hawkins T.D., Becker S., Pontasch S., Dove S., Hoegh-Guldberg O., Leggat W., Fisher P.L., Davy S.K. (2015). Differential Coral Bleaching-Contrasting the Activity and Response of Enzymatic Antioxidants in Symbiotic Partners under Thermal Stress. Comp. Biochem. Physiol. Part A Mol. Integr. Physiol..

[25] Lesser M.P. (1996). Elevated Temperatures and Ultraviolet Radiation Cause Oxidative Stress and Inhibit Photosynthesis in Symbiotic Dinoflagellates. Limnol. Oceanogr..

[26] Tchernov D., Gorbunov M.Y., De Vargas C., Yadav S.N., Milligant A.J., Häggblom M., Falkowski P.G. (2004). Membrane Lipids of Symbiotic Algae Are Diagnostic of Sensitivity to Thermal Bleaching in Corals. Proc. Natl. Acad. Sci. USA.

[27] Bienert G.P., Møller A.L.B., Kristiansen K.A., Schulz A., Møller I.M., Schjoerring J.K., Jahn T.P. (2007). Specific Aquaporins Facilitate the Diffusion of Hydrogen Peroxide across Membranes. J. Biol. Chem..

[28] Khorobrykh S., Havurinne V., Mattila H., Tyystjärvi E. (2020). Oxygen and ROS in Photosynthesis. Plants.

[29] Smith D.J., Suggett D.J., Baker N.R. (2005). Is Photoinhibition of Zooxanthellae Photosynthesis the Primary Cause of Thermal Bleaching in Corals?. Glob. Chang. Biol..

[30] Wooldridge S.A. (2013). Breakdown of the Coral-Algae Symbiosis: Towards Formalising a Linkage between Warm-Water Bleaching Thresholds and the Growth Rate of the Intracellular Zooxanthellae. Biogeosciences.

[31] Wooldridge S.A. (2009). A New Conceptual Model for the Warm-Water Breakdown of the Coralalgae Endosymbiosis. Mar. Freshw. Res..

[32] Rädecker N., Pogoreutz C., Gegner H.M., Cárdenas A., Roth F., Bougoure J., Guagliardo P., Wild C., Pernice M., Raina J.B. (2021). Heat Stress Destabilizes Symbiotic Nutrient Cycling in Corals. Proc. Natl. Acad. Sci. USA.

[33] Baker D.M., Freeman C.J., Wong J.C.Y., Fogel M.L., Knowlton N. (2018). Climate Change Promotes Parasitism in a Coral Symbiosis. ISME J..

[34] Wiedenmann J., D’Angelo C., Smith E.G., Hunt A.N., Legiret F.E., Postle A.D., Achterberg E.P. (2013). Nutrient Enrichment Can Increase the Susceptibility of Reef Corals to Bleaching. Nat. Clim. Chang..

[35] Hawkins T.D., Bradley B.J., Davy S.K. (2013). Nitric Oxide Mediates Coral Bleaching through an Apoptotic-like Cell Death Pathway: Evidence from a Model Sea Anemone-Dinoflagellate Symbiosis. FASEB J..

[36] Hawkins T.D., Davy S.K. (2012). Nitric Oxide Production and Tolerance Differ among *Symbiodinium* Types Exposed to Heat Stress. Plant Cell Physiol..

[37] Perez S., Weis V. (2006). Nitric Oxide and Cnidarian Bleaching: An Eviction Notice Mediates Breakdown of a Symbiosis. J. Exp. Biol..

[38] Trapido-Rosenthal H., Zielke S., Owen R., Buxton L., Boeing B., Bhagooli R., Archer J. (2005). Increased Zooxanthellae Nitric Oxide Synthase Activity Is Associated With Coral Bleaching. Biol. Bull..

[39] Bouchard J.N., Yamasaki H. (2008). Heat Stress Stimulates Nitric Oxide Production in *Symbiodinium microadriaticum*: A Possible Linkage between Nitric Oxide and the Coral Bleaching Phenomenon. Plant Cell Physiol..

[40] Hawkins T.D., Davy S.K. (2013). Nitric Oxide and Coral Bleaching: Is Peroxynitrite Generation Required for Symbiosis Collapse?. J. Exp. Biol..

[41] Hawkins T.D., Krueger T., Wilkinson S.P., Fisher P.L., Davy S.K. (2015). Antioxidant Responses to Heat and Light Stress Differ with Habitat in a Common Reef Coral. Coral Reefs.

[42] Hawkins T.D., Krueger T., Becker S., Fisher P.L., Davy S.K. (2014). Differential Nitric Oxide Synthesis and Host Apoptotic Events Correlate with Bleaching Susceptibility in Reef Corals. Coral Reefs.

[43] Jury C.P., Boeing B.M., Trapido-Rosenthal H., Gates R.D., Toonen R.J. (2022). Nitric Oxide Production Rather than Oxidative Stress and Cell Death Is Associated with the Onset of Coral Bleaching in *Pocillopora acuta*. PeerJ.

[44] Perez S.F. (2007). Exploring the Cellular Mechanisms of Cnidarian Bleaching in the Sea Anemone *Aiptasia pallida*. Doctoral Thesis.

[45] Dungan A.M., Maire J., Perez-Gonzalez A., Blackall L.L., Van Oppen M.J.H. (2022). Lack of Evidence for the Oxidative Stress Theory of Bleaching in the Sea Anemone, *Exaiptasia diaphana*, under Elevated Temperature. Coral Reefs.

[46] Dungan A.M., Hartman L.M., Blackall L.L., van Oppen M.J.H. (2022). Exploring Microbiome Engineering as a Strategy for Improved Thermal Tolerance in *Exaiptasia diaphana*. J. Appl. Microbiol..

[47] Puntin G., Craggs J., Hayden R., Engelhardt K., McIlroy S., Sweet M., Baker D.M., Ziegler M. (2022). The Reef-Building Coral *Galaxea fascicularis*: A New Model System for Coral Symbiosis. Coral Reefs.

[48] Weis V.M., Davy S.K., Hoegh-Guldberg O., Rodriguez-Lanetty M., Pringle J.R. (2008). Cell Biology in Model Systems as the Key to Understanding Corals. Trends Ecol. Evol..

[49] Tortorelli G., Belderok R., Davy S.K., Mcfadden G.I., van Oppen M.J.H., Levy O. (2020). Host Genotypic Effect on Algal Symbiosis Establishment in the Coral Model, the Anemone *Exaiptasia diaphana*, From the Great Barrier Reef. Front. Mar. Sci..

[50] Hartman L.M., van Oppen M.J.H., Blackall L.L. (2020). The Effect of Thermal Stress on the Bacterial Microbiome of *Exaiptasia diaphana*. Microorganisms.

[51] Hume B.C.C., D’Angelo C., Smith E.G., Stevens J.R., Burt J., Wiedenmann J. (2015). *Symbiodinium thermophilum* sp. nov., a Thermotolerant Symbiotic Alga Prevalent in Corals of the World’s Hottest Sea, the Persian/Arabian Gulf. Sci. Rep..

[52] Hume B., D’Angelo C., Burt J., Baker A.C., Riegl B., Wiedenmann J. (2013). Corals from the Persian/Arabian Gulf as Models for Thermotolerant Reef-Builders: Prevalence of Clade C3 *Symbiodinium*, Host Fluorescence and Ex Situ Temperature Tolerance. Mar. Pollut. Bull..

[53] Hume B.C.C., Smith E.G., Ziegler M., Warrington H.J.M., Burt J.A., LaJeunesse T.C., Wiedenmann J., Voolstra C.R. (2019). SymPortal: A Novel Analytical Framework and Platform for Coral Algal Symbiont next-Generation Sequencing ITS2 Profiling. Mol. Ecol. Resour..

[54] Dungan A.M., Hartman L., Tortorelli G., Belderok R., Lamb A.M., Pisan L., Mcfadden G.I., Blackall L.L., Van Oppen M.J.H. (2020). *Exaiptasia diaphana* from the Great Barrier Reef: A Valuable Resource for Coral Symbiosis Research. Symbiosis.

[55] R Core Team (2022). R: A Language and Environment for Statistical Computing.

[56] Lavy A., Eyal G., Neal B., Keren R., Loya Y., Ilan M. (2015). A Quick, Easy and Non-Intrusive Method for Underwater Volume and Surface Area Evaluation of Benthic Organisms by 3D Computer Modelling. Methods Ecol. Evol..

[57] Escada-Rebelo S., Mora F.G., Sousa A.P., Almeida-Santos T., Paiva A., João R.-S. (2020). Fluorescent Probes for the Detection of Reactive Oxygen Species. Asian J. Androl..

[58] Mydlarz L.D., Palmer C.V. (2011). The Presence of Multiple Phenoloxidases in Caribbean Reef-Building Corals. Comp. Biochem. Physiol.-A Mol. Integr. Physiol..

[59] Wickham H. (2016). Ggplot2: Elegant Graphics for Data Analysis.

[60] Gundlach K.A., Watson G.M. (2019). The Effects of Symbiotic State and Nutrient Availability on the Cnidom in the Model Sea Anemone, *Exaiptasia diaphana*. Mar. Biol..

[61] Sorek M., Schnytzer Y., Waldman Ben-Asher H., Caspi V.C., Chen C.S., Miller D.J., Levy O. (2018). Setting the Pace: Host Rhythmic Behaviour and Gene Expression Patterns in the Facultatively Symbiotic Cnidarian *Aiptasia* Are Determined Largely by *Symbiodinium*. Microbiome.

[62] Herrera M., Klein S.G., Schmidt-Roach S., Campana S., Cziesielski M.J., Chen J.E., Duarte C.M., Aranda M. (2020). Unfamiliar Partnerships Limit Cnidarian Holobiont Acclimation to Warming. Glob. Chang. Biol..

[63] Matthews J.L., Sproles A.E., Oakley C.A., Grossman A.R., Weis V.M., Davy S.K. (2016). Menthol-Induced Bleaching Rapidly and Effectively Provides Experimental Aposymbiotic Sea Anemones (*Aiptasia* sp.) for Symbiosis Investigations. J. Exp. Biol..

[64] Imlay J.A., Fridovich I. (1991). Assay of Metabolic Superoxide Production in *Escherichia coli*. J. Biol. Chem..

[65] Lardinois O.M., Mestdagh M.M., Rouxhet P.G. (1996). Reversible Inhibition and Irreversible Inactivation of Catalase in Presence of Hydrogen Peroxide. Biochim. Biophys. Acta.

[66] Saragosti E., Tchernov D., Katsir A., Shaked Y. (2010). Extracellular Production and Degradation of Superoxide in the Coral *Stylophora pistillata* and Cultured *Symbiodinium*. PLoS ONE.

[67] Armoza-Zvuloni R., Shaked Y. (2014). Release of Hydrogen Peroxide and Antioxidants by the Coral *Stylophora pistillata* to Its External Milieu. Biogeosciences.

[68] Armoza-Zvuloni R., Schneider A., Sher D., Shaked Y. (2016). Rapid Hydrogen Peroxide Release from the Coral *Stylophora pistillata* during Feeding and in Response to Chemical and Physical Stimuli. Sci. Rep..

[69] Higuchi T., Fujimura H., Arakaki T., Oomori T. Activities of Antioxidant Enzymes ( SOD and CAT ) in the Coral *Galaxea fascicularis* against Increased Hydrogen Peroxide Concentrations in Seawater. Proceedings of the 11th International Coral Reef Symposium.

[70] Roger L.M., Russo J.A., Jinkerson R.E., Giraldo J.P., Lewinski N.A. (2022). Engineered Nanoceria Alleviates Thermally Induced Oxidative Stress in Free-Living *Breviolum minutum* (Symbiodiniaceae, Formerly Clade B). Front. Mar. Sci..

[71] Setter R.O., Franklin E.C., Mora C. (2022). Co-Occurring Anthropogenic Stressors Reduce the Timeframe of Environmental Viability for the World’s Coral Reefs. PLoS Biol..

[72] van Oppen M.J.H., Oliver J.K., Putnam H.M., Gates R.D. (2015). Building Coral Reef Resilience through Assisted Evolution. Proc. Natl. Acad. Sci. USA.

[73] Chan W.Y., Peplow L.M., van Oppen M.J.H. (2019). Interspecific Gamete Compatibility and Hybrid Larval Fitness in Reef-Building Corals: Implications for Coral Reef Restoration. Sci. Rep..

[74] Chakravarti L.J., Beltran V.H., van Oppen M.J.H. (2017). Rapid Thermal Adaptation in Photosymbionts of Reef-Building Corals. Glob. Chang. Biol..

[75] Buerger P., Alvarez-Roa C., Coppin C.W., Pearce S.L., Chakravarti L.J., Oakeshott J.G., Edwards O.R., van Oppen M.J.H. (2020). Heat-Evolved Microalgal Symbionts Increase Coral Bleaching Tolerance. Sci. Adv..

[76] Rosado P.M., Leite D.C.A., Duarte G.A.S., Chaloub R.M., Jospin G., Nunes da Rocha U., Saraiva J.P., Dini-Andreote F., Eisen J.A., Bourne D.G. (2019). Marine Probiotics: Increasing Coral Resistance to Bleaching through Microbiome Manipulation. ISME J..

[77] Doering T., Wall M., Putchim L., Rattanawongwan T., Schroeder R., Hentschel U., Roik A. (2021). Towards Enhancing Coral Heat Tolerance: A “ Microbiome Transplantation ” Treatment Using Inoculations of Homogenized Coral Tissues. Microbiome.

[78] Santoro E.P., Borges R.M., Espinoza J.L., Freire M., Messias C.S., Villela H.D., Pereira L.M., Vilela C.L., Rosado J.G., Cardoso P.M. (2021). Coral Microbiome Manipulation Elicits Metabolic and Genetic Restructuring to Mitigate Heat Stress and Evade Mortality. Sci. Adv..

[79] Maire J., van Oppen M.J.H. (2022). A Role for Bacterial Experimental Evolution in Coral Bleaching Mitigation?. Trends Microbiol..

[80] Dungan A.M., Bulach D., Lin H., van Oppen M.J.H., Blackall L.L. (2021). Development of a Free Radical Scavenging Bacterial Consortium to Mitigate Oxidative Stress in Cnidarians. Microb. Biotechnol..

[81] Lajeunesse T.C., Bhagooli R., Hidaka M., DeVantier L., Done T., Schmidt G., Fitt W., Hoegh-Guldberg O. (2004). Closely Related *Symbiodinium* spp. Differ in Relative Dominance in Coral Reef Host Communities across Environmental, Latitudinal and Biogeographic Gradients. Mar. Ecol. Ser..

[82] Bongaerts P., Sampayo E.M., Bridge T.C.L., Ridgway T., Vermeulen F., Englebert N., Webster J.M., Hoegh-Guldberg O. (2011). *Symbiodinium* Diversity in Mesophotic Coral Communities on the Great Barrier Reef: A First Assessment. Mar. Ecol. Prog. Ser..

[83] Veron J.E.N., Stafford-Smith M.G., Turak E., DeVantier L.M. Corals of the World. http://www.coralsoftheworld.org/species_factsheets/species_factsheet_summary/galaxea-fascicularis/?version=0.01.

