# Supplementary material for: Comparing the Role of ROS and RNS in the Thermal Stress Response of Two Cnidarian Models, Exaiptasia diaphana and Galaxea fascicularis
Source: Antioxidants (Basel). 2023 May 6;12(5):1057. doi: 10.3390/antiox12051057 (PMC10215338; doi:10.3390/antiox12051057)
Supplement: Supplementary file 1 [file antioxidants-12-01057-s001.zip › antioxidants-2281009-supplementary.pdf]

Supplementary Material:

Comparing the role of ROS and RNS in the thermal stress response of two cnidarian models, *Exaiptasia diaphana* and *Galaxea fascicularis*

Talisa Doering [1,*], Justin Maire [1], Wing Yan Chan [1], Alexis Perez-Gonzalez [2,3], Luka Meyers [1], Rumi Sakamoto [1], Isini Buthgamuwa [1], Linda L. Blackall [1] and Madeleine J. H. van Oppen [1,4,*]

[1] School of Biosciences, The University of Melbourne, Parkville, VIC 3010, Australia
[2] Department of Microbiology and Immunology, The University of Melbourne at the Peter Doherty Institute of Infection and Immunity, Parkville, VIC 3010, Australia
[3] Melbourne Cytometry Platform, The University of Melbourne, Parkville, VIC 3010, Australia
[4] Australian Institute of Marine Science, Townsville MC, QLD 4810, Australia
* Correspondence: tdoring@student.unimelb.edu.au (T.D.); madeleine.van@unimelb.edu.au (M.J.H.v.O.); Tel.: +65-491-190045

Citation: Doering, T.; Maire, J.; Chan, W.Y.; Perez-Gonzalez, A.; Meyers, L.; Sakamoto, R.; Buthgamuwa, I.; Blackall, L.L.; van Oppen, M.J.H. Comparing the Role of ROS and RNS in the Thermal Stress Response of Two Cnidarian Models, *Exaiptasia diaphana* and *Galaxea fascicularis*. *Antioxidants* **2023**, *12*, 1057. https://doi.org/10.3390/antiox12051057

Academic Editors: Stanley Omaye, Antonio Box Centeno and Xavier Capo

Received: 28 February 2023
Revised: 24 April 2023
Accepted: 29 April 2023
Published: 6 May 2023

Copyright: © 2023 by the authors. Licensee MDPI, Basel, Switzerland. This article is an open access article distributed under the terms and conditions of the Creative Commons Attribution (CC BY) license (https://creativecommons.org/licenses/by/4.0/).

1. Supplemental Figures

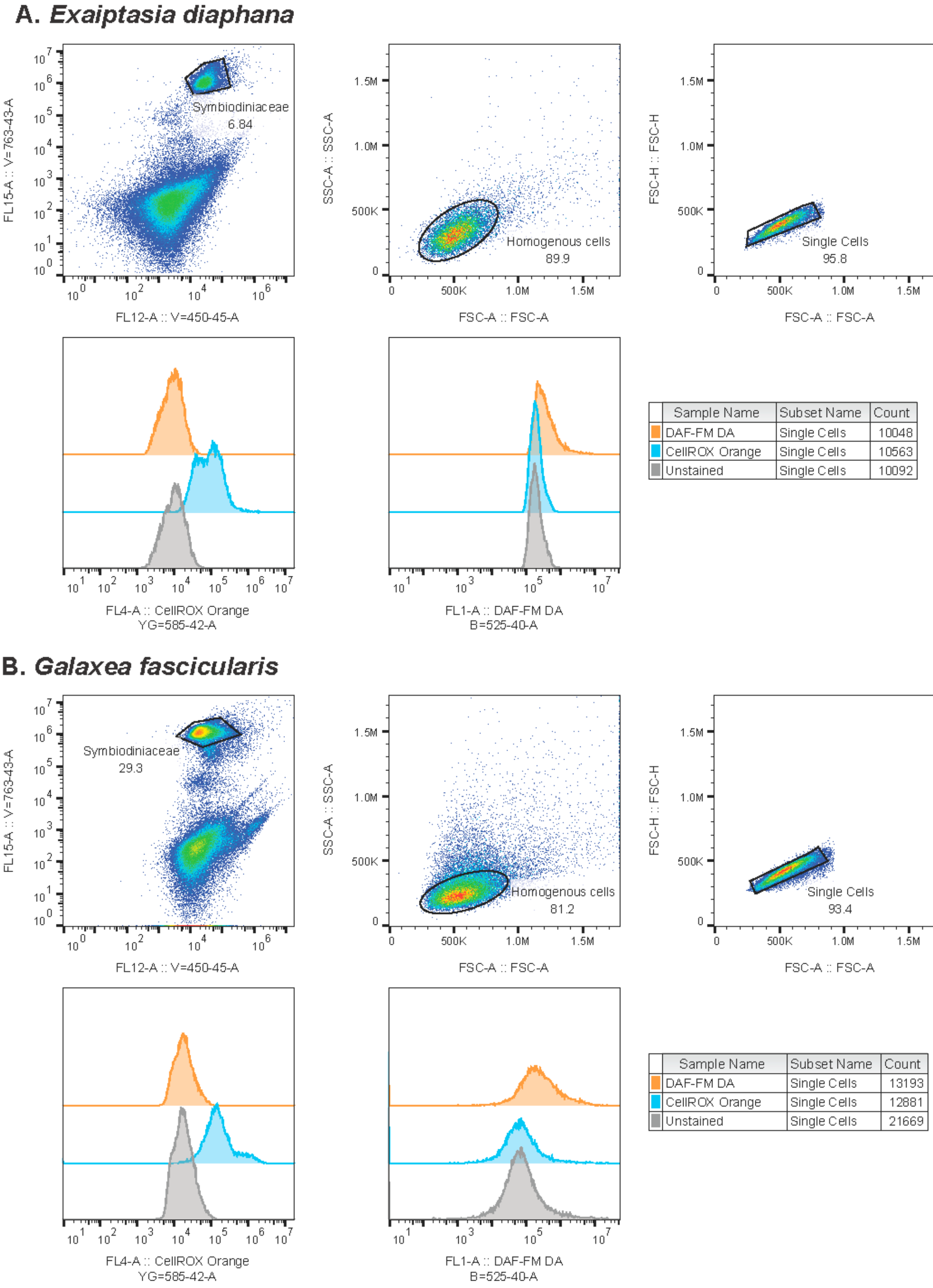

Supplemental Figure 1. ROS and RNS quantification in symbiodiniaceans freshly isolated from *Exaiptasia diaphana* (A) and *Galaxea fasiscularis* (B). The same gating procedure was followed for both organisms. To separate symbiodiniaceans from host cells, bacteria, and debris, we first selected events strongly excited by the 405 nm laser at emissions 450 ± 22.5 nm and 763 ± 21.5 nm ("Symbiodiniaceae" gate). An FSC-A/SSC-A plot was then used to select homogenous cells ("Homogenous" gate) and singlets were gated on an FSC-H/FSC-A plot ("Single cells" gate).

CellROX® Orange signal (ROS dye) was quantified as the median signal of stained minus unstained cells on single cells gated on previous plots with the 561 nm laser (emission 585 ± 21 nm). DAF-FM DA signal (RNS dye) was quantified as the median signal of stained minus unstained cells on single cells gated on previous plots with the 488 nm laser (emission 525 ± 20 nm). At least 10,000 symbiodiniacean singlets per sample were processed, except in samples where symbiodiniacean cell density was low because of advanced bleaching.

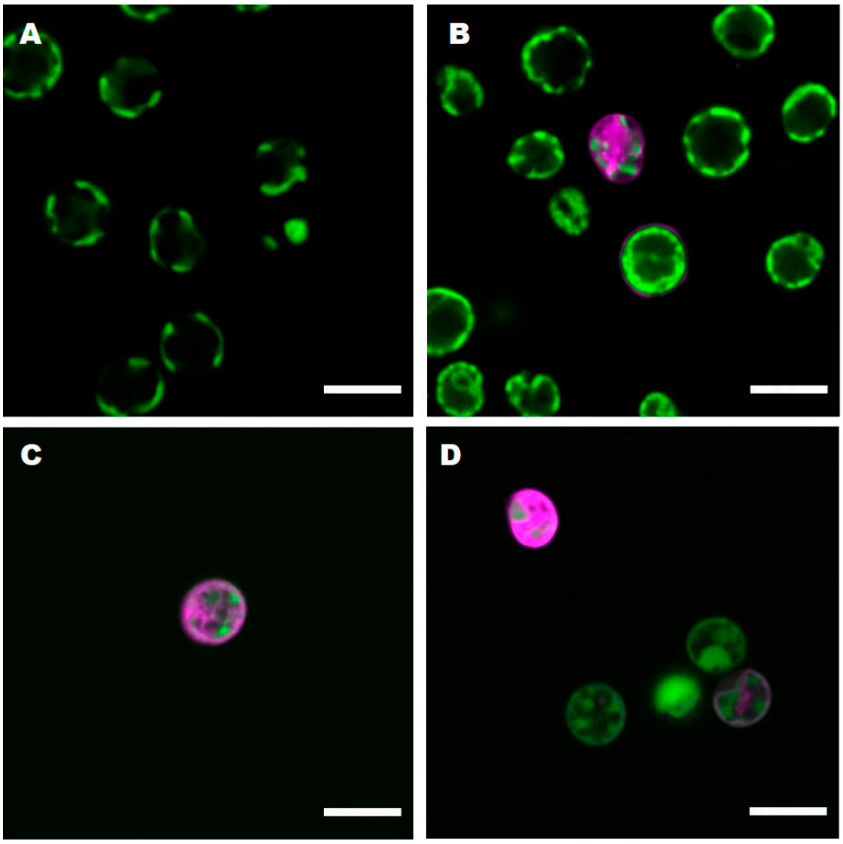

Supplemental Figure 2. Confocal laser scanning micrographs of **A:** cultured unstained *Breviolium minutum* (MMSF01, isolated from *Exaiptasia diaphana*), **B:** DAF-FM-DA stained *B. minutum*, **C-D:** DAF-FM-DA stained *E. diaphana* homogenate. Green indicates symbiodiniacean chl *a* autofluorescence and magenta indicates nitric oxide-dependent DAF-FM-DA fluorescence. DAF-FM-DA was detected using a 488 nm laser and its fluorescence was detected at emission 497-557 nm. A 568 nm laser was applied to detect chlorophyll a autofluorescence, that was detected at 600-710 nm. White scale bar = 10 µm.

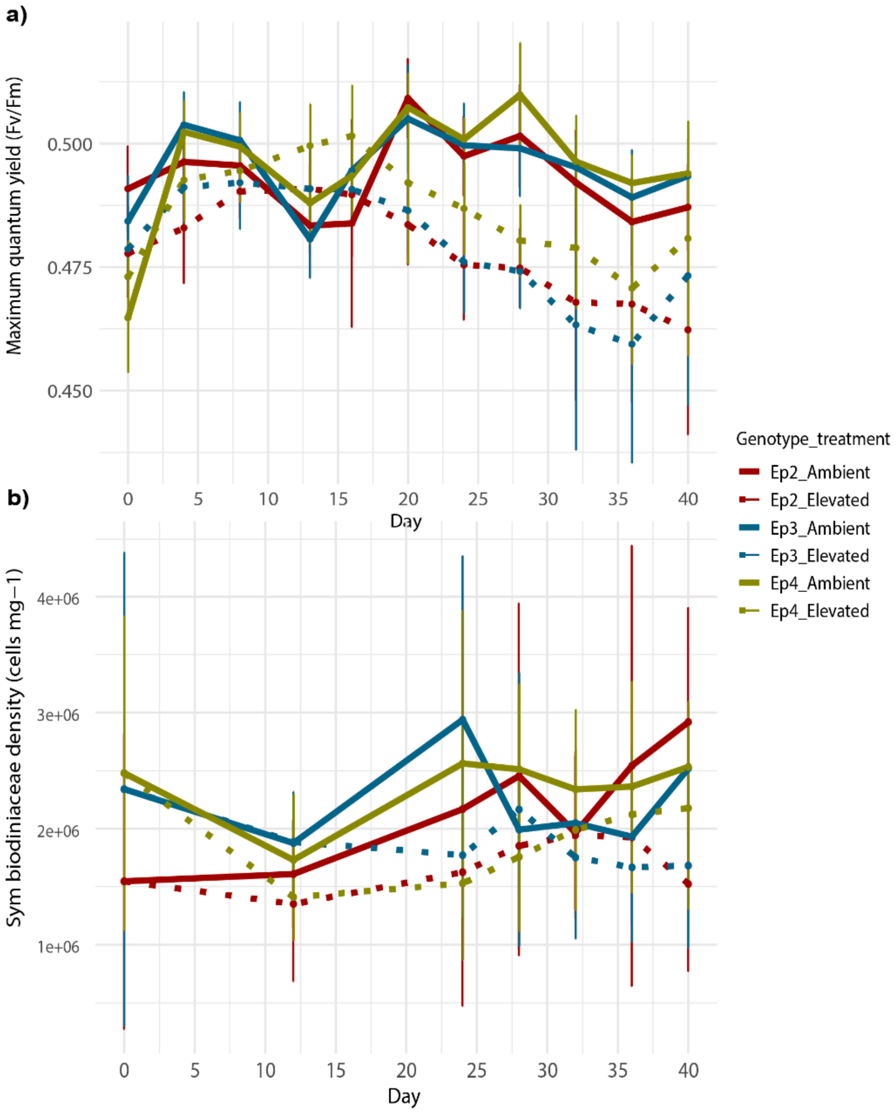

Supplemental Figure 3. a) Maximum quantum yield of symbiodiniaceans (F_v/F_m) (n=20 per genotype per treatment) and **b)** symbiodiniacean cell density (n=5) of *Exaiptasia diaphana*. Bars depict standard deviation.

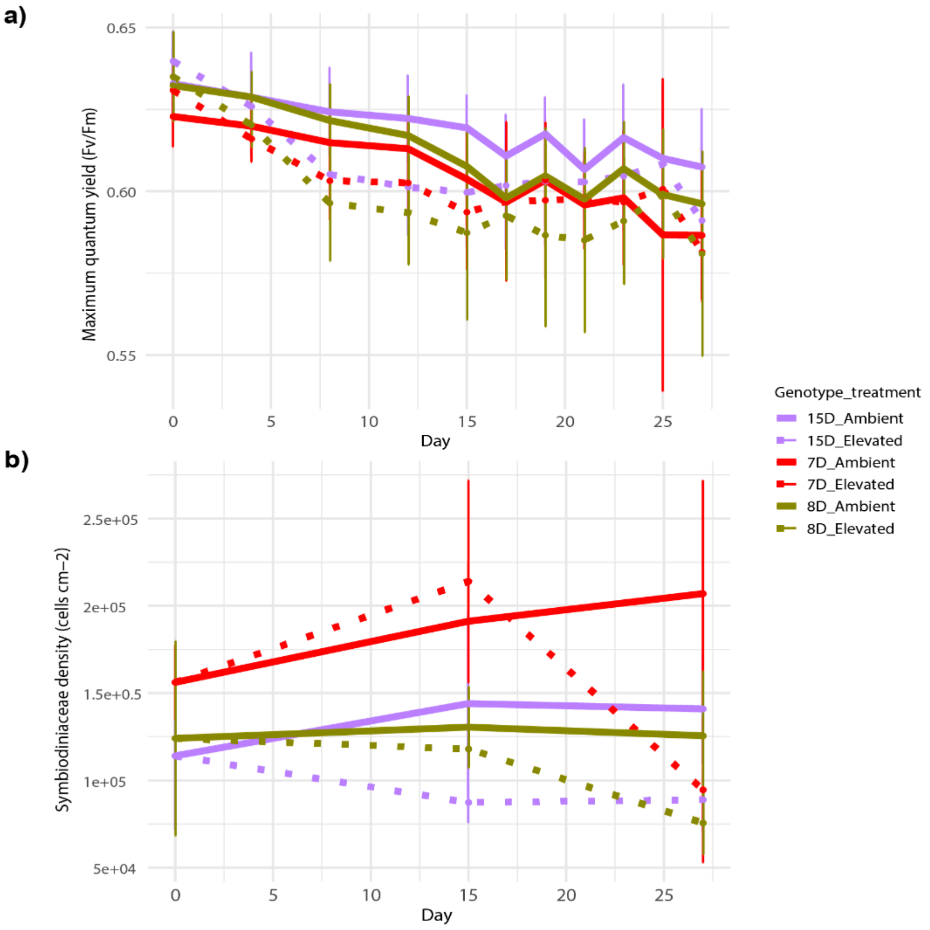

Supplemental Figure 4. a) Maximum quantum yield of symbiodiniaceans (F_v/F_m) (n=20 per colony per treatment) and **b)** symbiodiniacean cell density (n=5) of *Galaxea fascicularis*. Bars depict standard deviation.

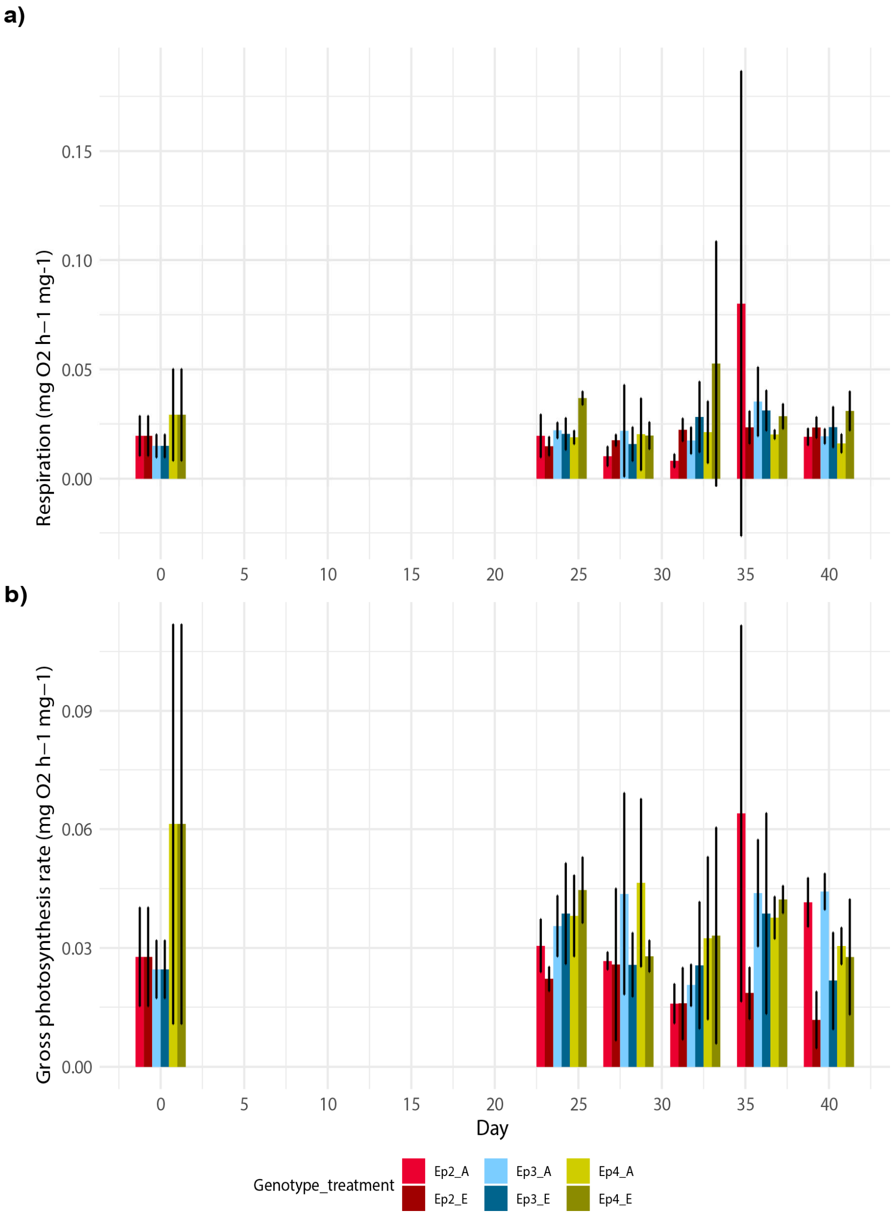

Supplemental Figure 5. a) Holobiont respiration rates (*n*=4 per genotype per treatment) and **b)** gross photosynthesis rates (*n*=4) of *Exaiptaisa diaphana*. Bars depict standard deviation.

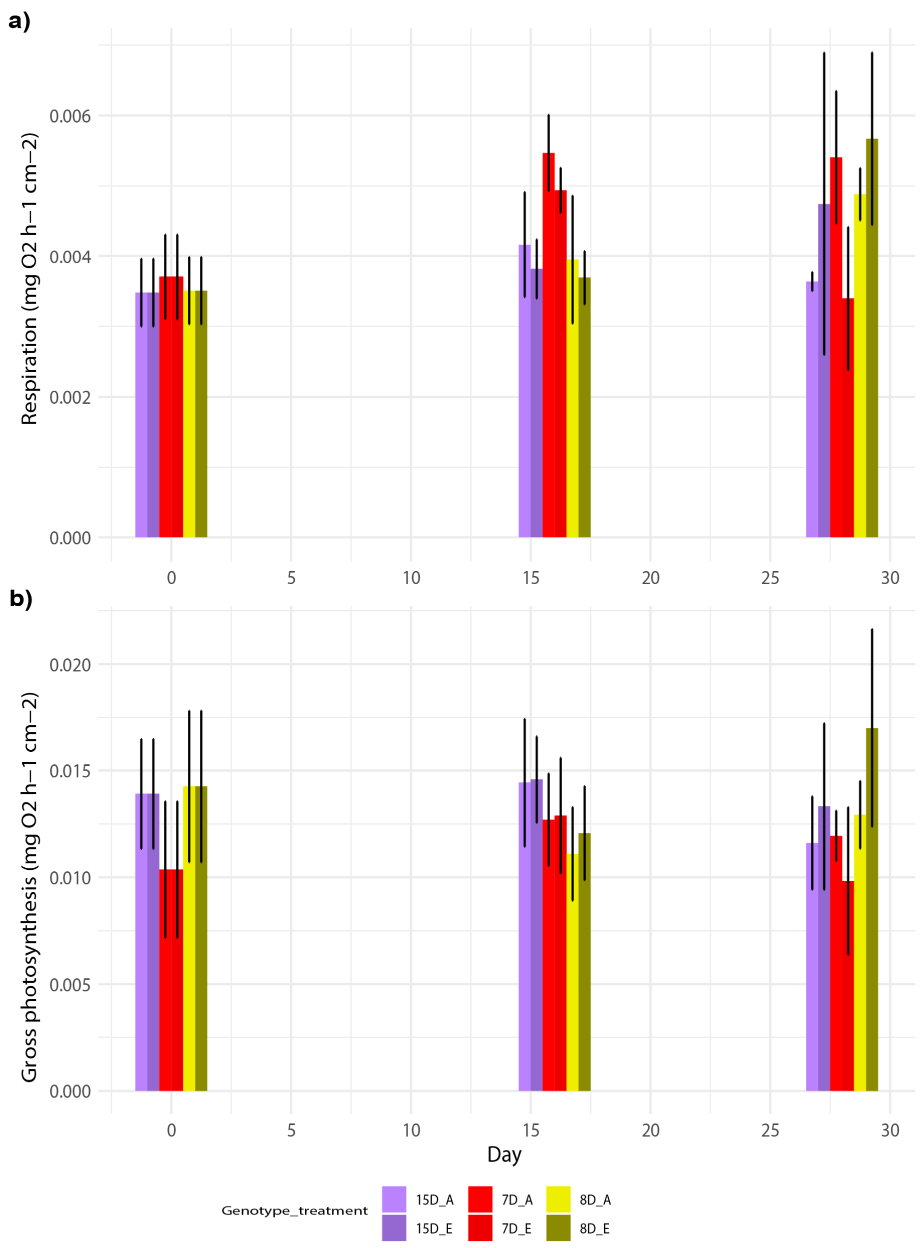

Supplemental Figure 6. a) Holobiont respiration rates (*n*=4 per colony per treatment) and **b)** gross photosynthesis rates (*n*=4) of *Galaxea fascicularis*. Bars depict standard deviation.

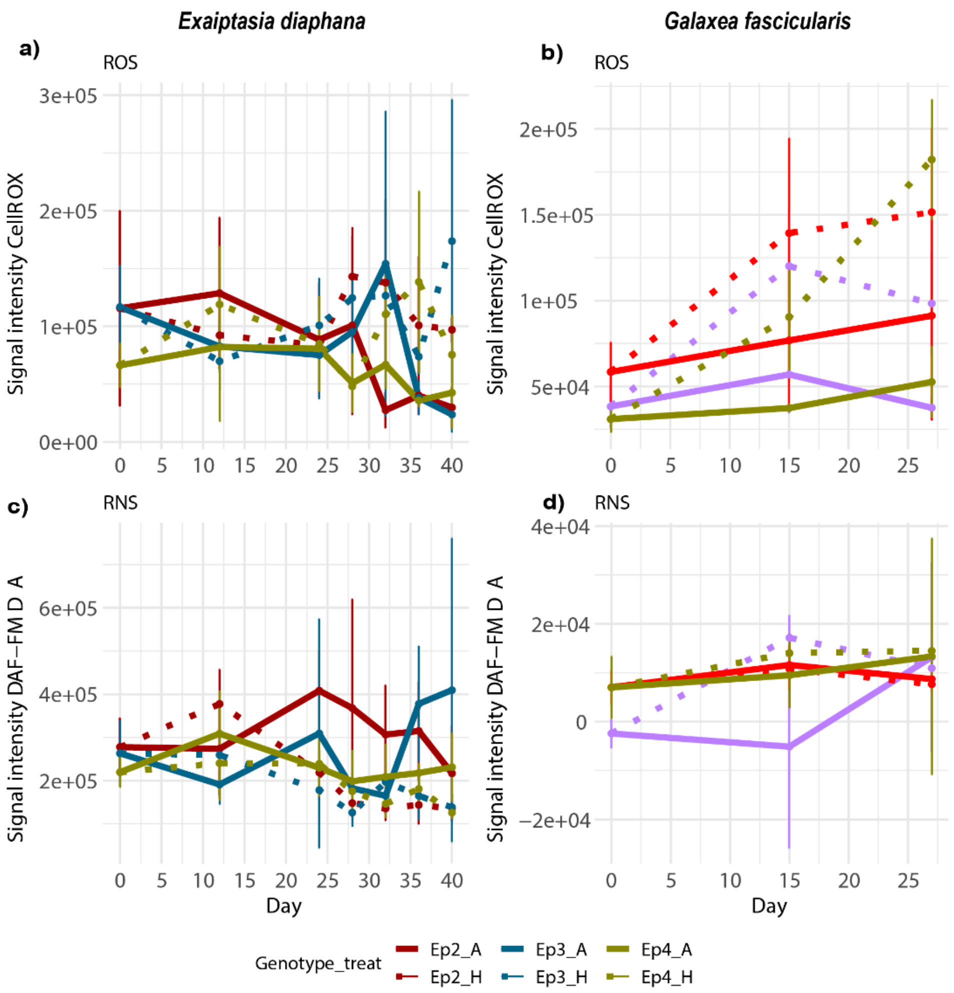

Supplemental Figure 7. Net ROS production of **a)** *Exaiptasia diaphana* and **b)** *Galaxea fascicularis* and net RNS production of **c)** *E. diaphana* and **d)** *G. fascicularis*. Net ROS was measured as signal intensity of CellROX stained symbiodiniacean cells. Net RNS was measured as signal intensity of DAF-FM DA stained symbiodiniacean cells. Signal intensities were normalized by subtracting the median signal of unstained cells to control for autofluorescence. Bars depict standard deviation. *N*=5 per sea anemone genotype or coral colony per temperature treatment.

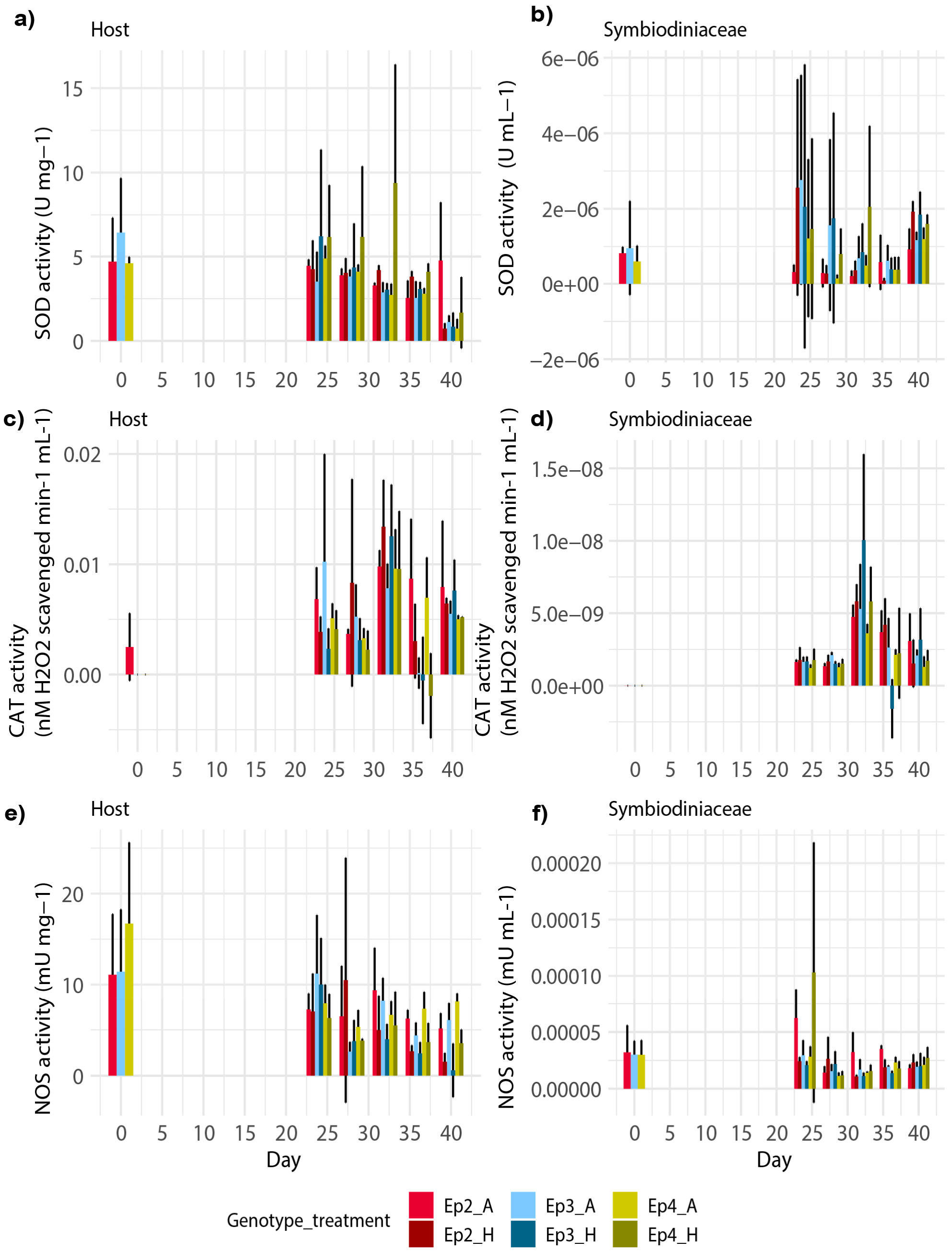

Supplemental Figure 8. Activities of SOD in a) host and b) symbiodiniaceans, of CAT in c) host and d) symbiodiniaceans and of NOS in e) host and f) symbiodiniacean compartments of *Exaiptasia diaphana*. Bars depict standard deviation. *N*=4 per genotype per temperature treatment.

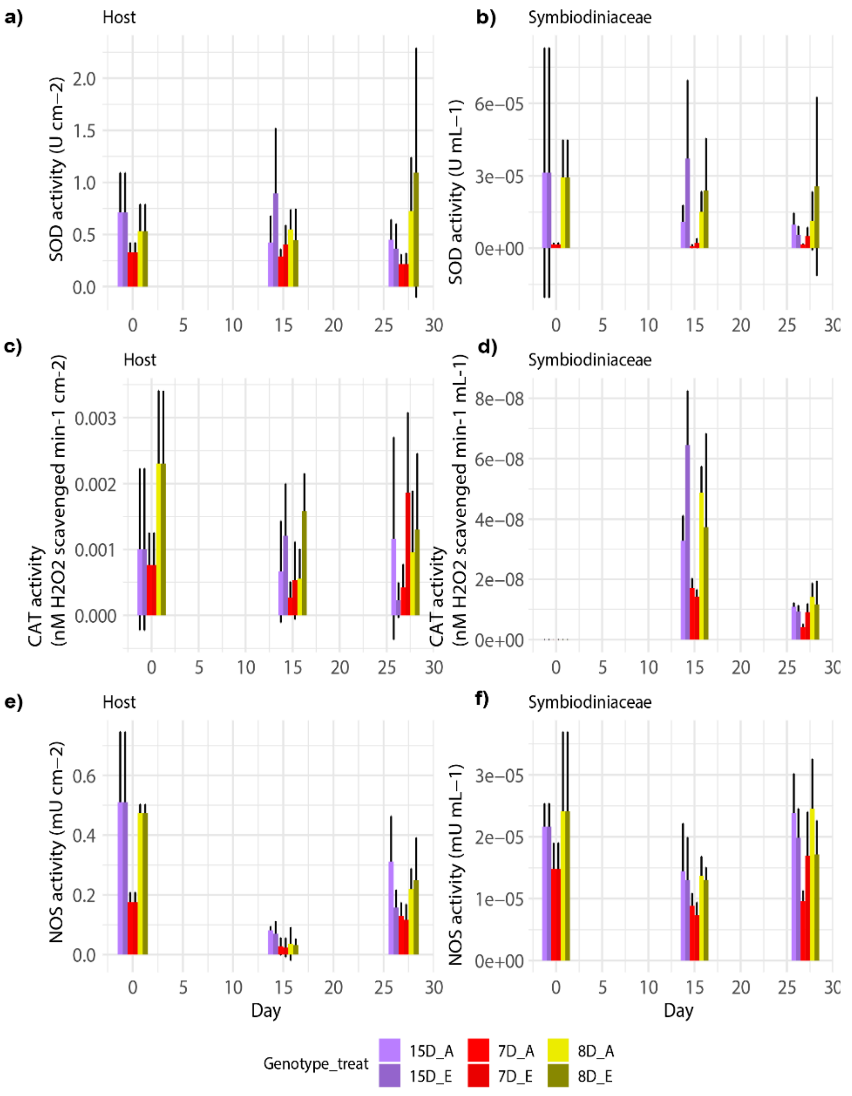

Supplemental Figure 9. Activities of SOD in a) host and b) Symbiodiniaceans, of CAT in c) host and d) symbiodiniaceans and of NOS in e) host and f) symbiodiniacean compartments of *Galaxea fascicularis*. Bars depict standard deviation. *N*=4 per colony per temperature treatment.

2. Supplemental Tables

Supplemental Table 1. Comparison of experimental settings and results of bleaching parameters of studies using GBR-sourced *Exaiptasia diaphana* genotypes. Results of bleaching parameters are displayed as relative changes of measured parameters in the elevated compared to the ambient temperature treatments at the last sampling timepoints of the experiments. Asterisks display statistical significance (*p*-value <0.001=***, <0.05=*).

						**Relative change in elevated versus ambient treatment at the last sampling timepoint**		
**Author**	**Study**	**Light conditions**	***Exaiptasia diaphana* genotype**	**Temperature profile in elevated treatment**	**Temperatu-re in ambient treatment**	**Maximum quantum yield**	**Symbiodin-iaceae density**	**net ROS production (if measured)**
This study		15-20 umol photons m-2 s-1	AIMS2, AIMS3, AIMS4	Day 0-24: increase of 0.25 °C/day to 32 °C (24 days), Day 24-40: constant 32°C (17 days)	Day 0-40: constant 26 °C	Significantly lower ***	No significant difference	Significantly higher ***, but this may be caused by a decrease in the ambient treatment
Dungan et al. 2022 [45]	Lack of evidence for the oxidative stress theory of bleaching in the sea anemone, *Exaiptasia diaphana,* under elevated temperature	15-20 μmol photons $m^{-2} s^{-1}$	AIMS3	Day 0-7: increase of 1 °C/day to 32 °C (6 days), Day 7-9: constant 32 °C (4 days), Day 10-12 increase of 1 °C/day to 34 °C (2 days), day 13: constant 34 °C (one day)	Day 0-13: constant 26 °C (14 days)	Significantly lower *	Significantly lower *	No significant difference
Dungan et al. 2022 [46]	Exploring microbiome engineering as a strateg	28 μmol photons $m^{-2} s^{-1}$	AIMS2, AIMS3, AIMS4	Day 0-7: constant 26 °C during inoculation (8 days), Day 8- 31: increase of 0.25 °C/day	Day 0-43: constant 26 °C (44 days)	Significantly lower *	Significantly lower * (for AIMS2 and AIMS3 only)	No significant difference

	y for improved thermal tolerance in *Exaiptasia diaphana*			to 31.5 °C (23 days), Day 31-43: constant 31.5 °C (12 days)				
Hartman et al. 2019 [50]	The Effect of Thermal Stress on the Bacterial Microbiome of *Exaiptasia diaphana*	31.8-33.8 μmol photon s m^{-2} s^{-1}	AIMS2	Day 0-14: increase of 1 °C/day to 33 °C (15 days)	Day 0-14: constant 26 °C	Significantly lower *	Significantly lower *	Not measured

Supplemental Table 2. Symbiodiniacean communities of the three coral colonies used in this study. All colonies were exclusively associated with *Durusdinium trenchii* (ITS2 profile D1-D4-D4c-D1ev-D10). #Non-profile sequences are sequences that have no ITS2 profile match.

Sample name	Sample category	Relative abundance (%)	
		Durusdinium trenchii **(D1-D4-D4c-D1ev-D10)**	**Non-profile sequences#**
G7A	Coral colony G7	92.2	7.8
G7B	Coral colony G7	92.3	7.7
G8A	Coral colony G8	92.1	7.9
G8B	Coral colony G8	92.7	7.3
G15A	Coral colony G15	92.8	7.2
G15B	Coral colony G15	92.1	7.9
NTPCR1	Negative control	0	0
NTPCR2	Negative control	0	0
ExtBlank	Negative control	0	0
